# Ischemia-free liver transplantation improves long-term outcomes in a 5-year follow-up study

**DOI:** 10.1016/j.jhepr.2025.101393

**Published:** 2025-03-12

**Authors:** Zehua Jia, Jiaxing Zhu, Jiayi Zhang, Jian Zhang, Changjun Huang, Niancun Zhang, Songming Li, Yuqi Dong, Yao Liu, Ping Zeng, Tielong Wang, Zhitao Chen, Yunhua Tang, Qiang Zhao, Maogen Chen, Yinghua Chen, Anbin Hu, Weiqiang Ju, Yi Ma, Dongping Wang, Xiaofeng Zhu, Andrea Schlegel, Tullius G. Stefan, Xiaoshun He, Zhiyong Guo

**Affiliations:** 1 Organ Transplant Center, The First Affiliated Hospital, Sun Yat-sen University, Guangzhou, China; 2 Guangdong Provincial Key Laboratory of Organ Medicine, Guangzhou, China; 3 Guangdong Provincial International Cooperation Base of Science and Technology (Organ Transplantation), Guangzhou, China; 4 State Key Laboratory of Ophthalmology, Zhongshan Ophthalmic Center, Sun Yat-sen University, Guangzhou, China; 5 Transplantation Center, Digestive Disease and Surgery Institute and Department of Immunology, Lerner Research Institute, Cleveland Clinic, Cleveland, OH, USA; 6 Division of Transplant Surgery, Brigham and Women's Hospital, Harvard Medical School, Boston 02115, MA, USA; 7 NHC Key Laboratory of Assisted Circulation, Sun Yat-sen University, Guangzhou, China

**Keywords:** Liver transplantation, Ischemia–reperfusion injury, Ischemia-free organ transplantation, Long-term outcomes

## Abstract

**Background & Aims:**

Ischemia-free liver transplantation (IFLT) is a novel technique designed to avoid ischemia–reperfusion injury (IRI). Here, we report the first detailed 5-year follow-up outcomes.

**Methods:**

We conducted a cohort study comparing long-term outcomes between IFLT and conventional liver transplantation (CLT) recipients of livers donated after brain death (DBD). The primary objective was to evaluate 5-year patient and graft survival. Additional endpoints included graft loss, biliary complications, rejection, infections, and liver-related laboratory tests. Subgroup analysis was performed to validate the generalizability of the results in patients with pre-transplant hepatocellular carcinoma (HCC).

**Results:**

A total of 168 patients were enrolled, with 38 patients in the IFLT group and 130 patients in the CLT group. Five-year patient survival (86.84% *vs*. 56.92%; hazard ratio [HR] 0.246, 95% confidence interval [CI] 0.098–0.620; *p* <0.01) and graft survival (84.61% *vs*. 56.92%; HR 0.307, 95% CI 0.131–0.719; *p* <0.01) rates were significantly improved in the IFLT group compared with the CLT group. In the multivariate analysis, IFLT emerged as an independent protective factor for 5-year patient survival (HR 0.246, 95% CI 0.098–0.620; *p* <0.01). Conversely, HCC before transplantation (HR 2.039, 95% CI 1.159–3.590; *p* <0.05), donor age (HR 1.022, 95% CI 1.001–1.040; *p* <0.05), and extended criteria donor (HR 2.088, 95% CI 1.215–3.590; *p* <0.01) were identified as independent risk factors for impaired 5-year patient survival. In patients with pre-transplant HCC, the 5-year overall survival rate of the IFLT group was also significantly higher than that of the CLT group after adjustment for HCC risk factors (82.35% *vs*. 42.03%; HR 0.249, 95% CI 0.074–0.831; *p* <0.05).

**Conclusions:**

Long-term (5-year) follow-up data demonstrate that the use of IFLT potentially improves both patient and graft survival, when compared with CLT, in transplantation of brain-dead donor livers.

**Impact and implications:**

Ischemia-free liver transplantation (IFLT) has emerged as a new approach designed to avoid IRI throughout all episodes of the transplant procedure. It has been confirmed that the use of IFLT can substantially reduce early-onset graft IRI-related complications. In this first 5-year follow-up study on the IFLT technique, we demonstrate that, compared with conventional liver transplantation, IFLT can potentially improve long-term patient and graft survival by reducing cancer recurrence. This new technique has the potential to change current clinical practice, particularly in the use of marginal grafts and in patients with HCC.

**Clinical Trials registration:**

chictr.org (ChiCTR-OPN-17012090).

## Highlights

• This is the first 5-year follow-up study to document critical components of long-term outcomes on the IFLT technique.
• Compared with conventional liver transplantation, IFLT significantly improved both 5-year patient and graft survival rates.
• IFLT reduces various long-term complications, including tumor recurrence.

## Graphical abstract

**Figure:**
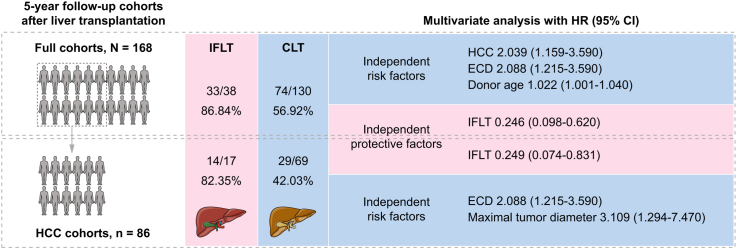

## Introduction

Liver transplantation represents the standard of care for end-stage liver diseases.^1^ Conventional liver transplantation (CLT) techniques inevitably involve ischemic periods during organ procurement, preservation, and implantation, which significantly impact short-term and long-term transplant outcomes as a consequence of ischemia–reperfusion injury (IRI).^2^^,^^3^

To mitigate the detrimental effects of IRI, various *ex situ* machine perfusion technologies, including hypothermic machine perfusion, hypothermic oxygenated perfusion (HOPE), normothermic machine perfusion (NMP), and subnormothermic machine perfusion (SNMP), have been used in liver transplantation.^4^ Recent randomized controlled trials (RCTs) have demonstrated that HOPE can reduce the incidence of non-anastomotic stricture (NAS)^5^ and >IIIb complications in transplantation of livers donated after circulatory death.^6^ In addition, the results of RCTs have shown that NMP can ameliorate the incidence of early allograft dysfunction (EAD).^7^^,^^8^ However, the grafts still experienced ischemic injury before and after *ex situ* machine perfusion, making IRI unavoidable under these conditions.

Ischemia-free liver transplantation (IFLT) has emerged as a new approach designed to avoid IRI by maintaining a continuous oxygenated blood supply throughout all episodes of the transplant procedure.^9^ Our comprehensive pathological, transcriptomic, and metabolomic analyses have demonstrated that IFLT can largely abrogate graft IRI.^10^ The results of the first-in-human trial have shown that the use of IFLT can substantially reduce graft IRI-related complications.^11^ Subsequent RCTs and retrospective studies further confirmed the safety and efficacy of IFLT.[12], [13], [14], [15] However, because of the short follow-up duration and limited sample sizes, evidence of the long-term benefits of IFLT is still lacking, and no 5-year follow-up data have been reported.

In the current study, we prospectively document critical components of long-term outcomes over a 5-year follow-up period in the same cohorts from our first-in-human IFLT trial.^11^

## Patients and methods

### Study, setting, and ethics

Our group conducted the first-in-human trial to assess the feasibility and safety of IFLT at The First Affiliated Hospital of Sun Yat-sen University between January 2017 and March 2019 (chictr.org: ChiCTR-OPN-17012090). Intraoperative and post-transplant care was performed according to standard protocols applied at our center. This was an investigator-initiated, single-center, prospective trial, and both cohorts were followed up for 5 years in this study. The protocol was approved by the Ethical Committee of The First Affiliated Hospital of Sun Yat-sen University, and all patients provided written informed consent.

### Participants

All patients receiving liver transplantation and fulfilling eligibility criteria during the study period were enrolled in this trial. All livers were procured from brain-dead donors and allocated by the China Organ Transplant Response System (COTRS) based on the emergency of diseases and waiting time.^16^ Allocation was non-randomized, and patients received IFLT based on the availability of NMP device disposables and perfusionists. Detailed eligibility criteria included the following: donation after brain death (DBD) donors aged >12 years whose organs were procured at our hospital were eligible for inclusion. Donation after cardiac death (DCD) donors were excluded. Adult recipients (>18 years) undergoing first liver-only transplantation in our hospital were included in the trial. Patients were excluded if they underwent combined organ transplantation, multi-visceral transplantation, split liver transplantation, or ABO-incompatible liver transplantation. All enrolled patients were informed of the procedural risks and provided written consent to receive IFLT.

### Trial interventions

In the IFLT group, donor livers underwent ischemia-free procurement under *in situ* NMP using the Liver Assist device (Organ Assist, Groningen, Netherlands); livers were preserved and assessed for viability under *ex situ* NMP. After the recipient’s hepatectomy, ischemia-free liver implantation (standard bicaval or piggyback technique) was performed under *in situ* NMP. Thus, all livers were procured, preserved, and implanted without interruption of normothermic, oxygenated blood supply.

In the CLT group, donor livers were procured using a standard *in situ* cold flushing procedure and preserved in 0–4 °C University of Wisconsin (UW) solution. After the removal of diseased livers, the donor livers were subsequently transplanted using the standard bicaval or piggyback technique.

Further details concerning study design, sample size calculation, surgical procedures, NMP protocols, and standards of perioperative care are described in detail in the original trial report and protocol.^11^

### Follow-up and outcomes

All patients enrolled in the original trial were followed up for 5 years. The primary objective of this study was to document 5-year overall patient survival and graft survival. Other endpoints included 5-year patient survival censored for tumor-related deaths, long-term infections, acute and chronic rejection, biliary complications,^17^ re-transplantation, and detailed causes of graft loss and patient death. In addition, we compared long-term liver-related laboratory test results between IFLT and CLT recipients, including alanine aminotransferase (ALT), aspartate aminotransferase (AST), total bilirubin (TBIL), alkaline phosphatase (ALP), gamma-glutamyltransferase (GGT), glucose (GLU), prealbumin (PA), and creatinine (CREA).

### Statistical analysis

Continuous variables were compared using the *t* test or the Mann–Whitney *U* test, whereas categorical variables were analyzed using the Chi-square test and Fisher’s exact test. Time-to-event outcomes are presented as Kaplan–Meier curves with hazard ratios (HRs) and *p* values calculated using multivariate Cox proportional hazards regression models. Baseline variables of donors and recipients that showed a univariate relationship with patient death (*p* <0.1) were entered into multivariate Cox proportional hazards regression models as covariates. All statistical analyses were based on two-sided tests, and results are reported with 95% confidence intervals (CIs). The significance level for the *p* value was set at 0.05. Statistical analysis was conducted using R 4.3.2 (R Foundation for Statistical Computing, Vienna, Austria).

## Results

### Patient characteristics

Of 412 donor livers allocated to our center from January 1, 2017, to March 12, 2019, a total of 168 patients (38 in the IFLT group and 130 in the CLT group) were included in this study. All patients completed a 5-year follow-up without any loss or withdrawal. Twenty-three patients in the CLT group and three patients in the IFLT group had died within 1 year. By the end of the observation period (year 5), an additional 33 patient deaths occurred in the CLT group and two patient deaths in the IFLT group (Fig. S1). The baseline characteristics of donors, recipients, and surgeries are detailed in Table S1 and the initial trial report,^11^ which were balanced between the IFLT and CLT groups.

### Five-year patient survival

**Fig. 1:**
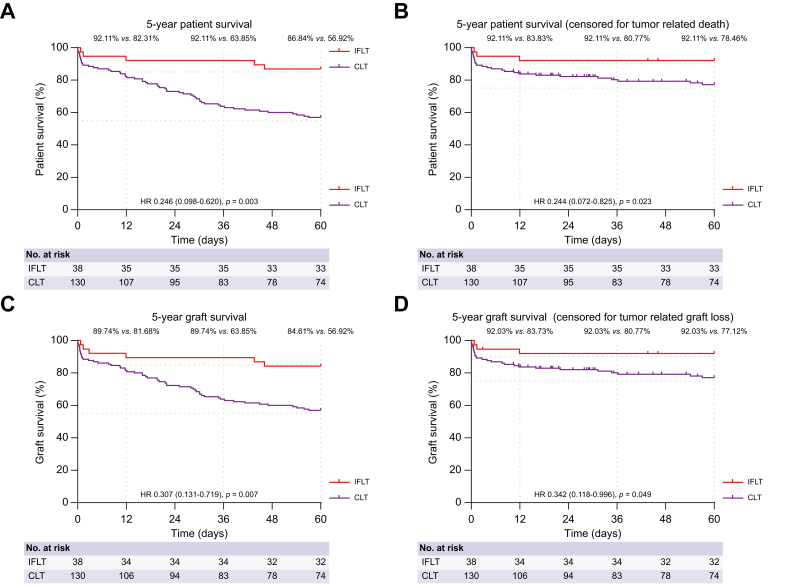
Five-year patient and graft survival in the IFLT and CLT groups. Time-to-event Kaplan–Meier curves for (A) 5-year patient survival, (B) patient survival censored for tumor-related death, (C) graft survival, and (D) graft survival censored for tumor-related graft loss after liver transplantation. The HR and *p* value in (A)–(D) were calculated using the Cox regression adjusted for donor (age and type) and recipient (HCC before transplantation) risk factors. Statistical significance was set at *p* <0.05. CLT, conventional liver transplantation; IFLT, ischemia-free liver transplantation; HR, hazard ratio.

The overall patient survival between the IFLT and CLT groups at 1, 3, and 5 years was 92.11% *vs*. 82.31%, 92.11% *vs*. 63.85%, and 86.84% *vs*. 56.92%, respectively (Fig. 1A). The 5-year overall survival rate was significantly higher in the IFLT group than in the CLT group after adjustment for donor (age and type) and recipient (hepatocellular carcinoma [HCC] before transplantation) risk factors (HR 0.246, 95% CI 0.098–0.620; *p* <0.01). The unadjusted result remained essentially the same (HR 0.252, 95% CI 0.101–0.628; *p* <0.01) (Table S2). Notably, there was a lower incidence of graft failure (0% *vs*. 8.46%; *p* = 0.138) and tumor recurrence (2.63% *vs*. 21.54%; *p* <0.01) in the IFLT group, representing the main explanation for superior patient survival. The incidence of infections, new-onset tumors, and other unforeseen events leading to patient death were comparable between the two groups (Table 1). In addition, the 5-year survival rate censored for tumor-related death was still significantly higher in the IFLT than in the CLT group (92.11% *vs*. 78.46%; HR 0.244, 95% CI 0.072–0.825; *p* <0.05) (Fig. 1B).

**Table 1:** Outcomes and complications.

Outcome parameter	IFLT (n = 38)	CLT (n = 130)	*p* value
Biliary complications, n (%)			0.63
Cholangitis	0	7 (5.38)	0.317
Non-anastomotic strictures	0	3 (2.31)	0.804
PTCD + ERCP + endoscopic stenting	0	1 (0.77)	>0.999
Long-term use of choleretic drugs	0	2 (1.54)	>0.999
Anastomotic strictures	5 (13.16)	20 (15.38)	0.734
Bile leak	1 (2.63)	4 (3.08)	>0.999
Biliary stone	2 (5.26)	6 (4.62)	0.977
Acute rejection, n (%)	7 (18.42)	39 (30.00)	0.159
Infection, n (%)			
Pneumonia	4 (10.53)	23 (17.69)	0.290
Sepsis	3 (7.89)	9 (6.92)	0.878
Other infection	5 (13.16)	11 (8.46)	0.474
Re-transplantation, n (%)	1 (2.63)	1 (0.77)	0.402
Cause of graft loss, n (%)			0.078
Liver-related graft loss	2 (5.26)	39 (30.00)	0.004
Primary non-function	0	4 (3.08)	0.575
Hepatic artery thrombosis	0	7 (5.38)	0.317
Tumor recurrence	1 (2.63)	27 (20.77)	0.008
Rejection	1 (2.63)	1 (0.77)	0.402
Non-liver-related graft loss	4 (10.53)	17 (13.08)	0.889
Cause of patient death, n (%)			0.007
Graft failure	0 (0)	11 (8.46)	0.138
Tumor recurrence	1 (2.63)	28 (21.54)∗	0.007
New-onset tumor	1 (2.63)	0	0.226
Sepsis/infection	2 (5.26)	12 (9.23)	0.656
Miscellaneous†	1 (2.63)	5 (3.85)	>0.999

The Chi-square test or Fisher’s exact test was used to calculated *p* values, with statistical significance defined as *p* <0.05.

CLT, conventional liver transplantation; ERCP, endoscopic retrograde cholangiopancreatography; HCC, hepatocellular carcinoma; IFLT, ischemia-free liver transplantation; PTCD, percutaneous transhepatic cholangiography.

∗ One patient underwent re-transplantation because of hepatic artery thrombosis (the cause of graft loss) and subsequently succumbed to HCC recurrence (the cause of patient death). In total, 25 patients died from HCC recurrence, one from hepatic epithelioid hemangioendothelioma recurrence, and two from cholangiocarcinoma recurrence.

† Miscellaneous included one case of heart failure, one vehicle accident, one head trauma, one suicide in the CLT group, and one case of acute pulmonary embolism in the IFLT group. The cause of death of one patient in the CLT group was unclear, as the patient’s family refused to provide further information.

### Five-year graft survival

The overall graft survival comparing IFLT and CLT recipients at 1, 3, and 5 years was 89.74% *vs*. 81.68%, 89.74% *vs*. 63.85%, and 84.61% *vs*. 56.92%, respectively (Fig. 1C). The 5-year graft survival rate was significantly higher in the IFLT group than in the CLT group after adjustment for donor and recipient risk factors (HR 0.307, 95% CI 0.131–0.719; *p* <0.01). The unadjusted result remained essentially the same (HR 0.308, 95% CI 0.133–0.716; *p* <0.01) (Table S2). IFLT recipients had a lower incidence of liver-related graft loss (5.26% *vs*. 30.00%; *p* <0.01). In the IFLT group, liver-related graft loss was attributed to tumor recurrence (n = 1) and chronic rejection (n = 1). In the CLT group, liver-related graft loss occurred because of primary non-function (PNF) (n = 4), hepatic artery thrombosis (HAT) (n = 7), tumor recurrence (n = 27), and chronic rejection (n = 1). The incidence of non-liver-related graft loss was comparable between the two groups (10.53% *vs*. 13.08%; *p* = 0.889). Finally, one patient in each group underwent re-transplantation (Table 1). In addition, the 5-year graft survival rate censored for tumor-related graft loss was still significantly superior in the IFLT compared with the CLT group (92.03% *vs*. 77.12%; HR 0.342, 95% CI 0.118–0.996; *p* = 0.049) (Fig. 1D).

### Risk factors of patient survival

Risk factors associated with patient death were further explored. In the univariate analysis, donor age, donor types (extended *vs*. standard criteria), transplant technique (IFLT *vs*. CLT), and recipients’ principal diagnosis (HCC *vs*. decompensated cirrhosis) affected patient survival (*p* <0.05). Then, risk factors with a *p* value <0.1 in the univariate analysis were included in the multivariate analysis. The use of the IFLT technique was an independent protective factor for long-term patient survival (HR 0.246, 95% CI 0.098–0.620; *p* <0.01). Donor age (HR 1.022, 95% CI 1.001–1.040; *p* <0.05), HCC before transplantation (HR 2.039, 95% CI 1.159–3.590; *p* <0.05), and extended criteria donor (HR 2.088, 95% CI 1.215–3.590; *p* <0.01) were independent risk factors for long-term patient survival (Table 2).

**Table 2:** Univariate and multivariate analyses of factors impacting patient survival.

	Univariate analysis		Multivariate analysis∗	
	HR (95% CI)	*p* value	HR (95% CI)	*p* value
**Donor characteristics**				
Age (years)	1.024 (1.004–1.045)	0.022	1.022 (1.001–1.040)	0.040
Sex				
Male	Reference	NA		
Female	0.930 (0.519–1.665)	0.806		
BMI (kg/m^2^)	1.038 (0.933–1.154)	0.492		
Causes of death				
Head trauma	Reference	NA		
Anoxia	0.399 (0.098–1.634)	0.201		
Cerebrovascular accident	1.379 (0.834–2.278)	0.210		
Miscellaneous	1.975 (0.716–5.448)	0.189		
Donor risk index	2.532 (0.822–7.804)	0.106		
Type				
Standard criteria donor	Reference	NA		
Extended criteria donor†	2.044 (1.204–3.469)	0.008	2.088 (1.215–3.590)	0.008

**Recipient characteristics**				
Age (years)	1.010 (0.985–1.035)	0.443		
Sex				
Male	Reference	NA		
Female	0.521 (0.163–1.663)	0.271		
HBV infection				
Negative	Reference	NA		
Positive	1.250 (0.594–2.629)	0.557		
Waiting time	0.997 (0.991–1.003)	0.334		
Laboratory MELD score‡	1.004 (0.973–1.035)	0.821		
Transplant techniques				
CLT	Reference	NA		
IFLT	0.252 (0.101–0.628)	0.003	0.246 (0.098–0.620)	0.003
Principal diagnosis				
Decompensate cirrhosis	Reference	NA		
HCC	2.487 (1.433–4.319)	0.001	2.039 (1.159–3.590)	0.013
Liver failure	0.517 (0.222–1.201)	0.125		
Miscellaneous	2.062 (0.645–6.597)	0.223		

**Operation characteristics**				
Anhepatic phase (min)	0.999 (0.985–1.013)	0.905		
Recipient operation time (min)				
≤600	Reference	NA		
>600	1.455 (0.527–4.010)	0.469		
Blood loss (ml)				
≤2000	Reference	NA		
>2000	1.255 (0.757–2.080)	0.378		
Intraoperative use of RBCs (ml)				
≤2000	Reference	NA		
>2000	1.398 (0.757–2.580)	0.285		
Intraoperative use of FFP (ml)				
≤2000	Reference	NA		
>2000	0.926 (0.523–1.639)	0.792		

Univariate and multivariate analyses were performed using Cox regression. Statistical significance was set at *p* < 0.05.

CLT, conventional liver transplantation; FFP, fresh frozen plasma; HCC, hepatocellular carcinoma; HR, hazard ratio; IFLT, ischemia-free liver transplantation; INR, international normalized ratio; MELD, model for end-stage liver disease; NA, not applicable; NMP, normothermic machine perfusion; RBC, red blood cell; UNOS, United Network for Organ Sharing.

∗ Variables with a *p* value <0.1 in the univariate analysis were included in the multivariate analysis.

† Extended criteria donor was defined as meeting at least one of the following criteria: (1) donor age >60 years; (2) hypernatremia (serum Na^+^ >165 mmol/L); (3) >30% macrovesicular steatosis by biopsy; (4) donor serum aspartate aminotransferase or alanine aminotransferase >1000 IU/L or total bilirubin >3 mg/dl before procurement; or (5) cold ischemia time ≥12 h. Detailed specific criteria fulfilled by each extended criteria donor in this study have been provided in the published article.^11^

‡ Laboratory MELD score was calculated using the formula 3.8[Ln serum bilirubin (mg/dl)] + 11.2[Ln INR] + 9.6[Ln serum creatinine (mg/dl)] + 6.4, as described by the UNOS for liver transplant prioritization.

### Patient and graft survival in patients with HCC

It was found that HCC before transplantation was a critical independent factor affecting patient survival. Next, we detailed our analysis considering characteristics that define the tumor stage.

**Fig. 2:**
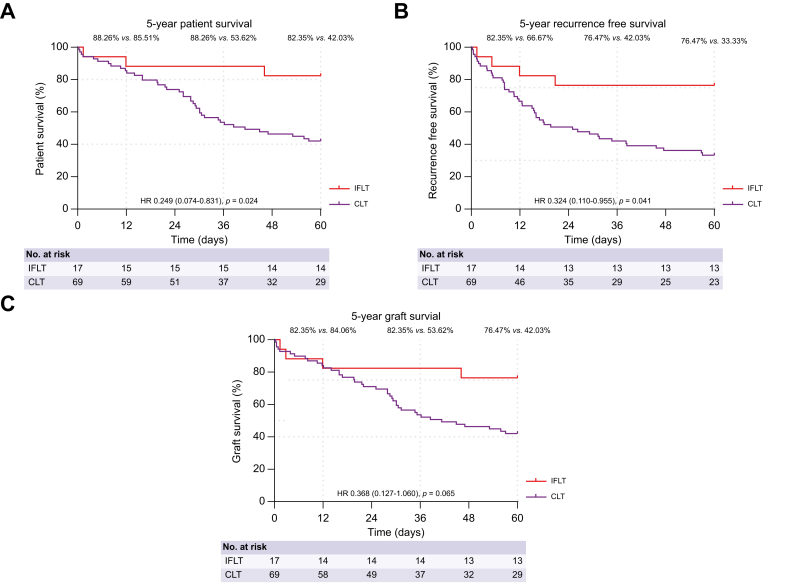
Five-year survival after liver transplantation in patients with HCC. Time-to-event Kaplan–Meier curves for (A) 5-year patient survival, (B) recurrence-free survival, and (C) graft survival after liver transplantation in patients with HCC. The HR and *p* value in (A)–(C) were calculated using the Cox regression adjusted for donor (type) and recipient (maximal tumor diameter, number of tumors, and tumor stages outside of the Milan and UCSF criteria) risk factors. Statistical significance was set at *p* <0.05. CLT, conventional liver transplantation; IFLT, ischemia-free liver transplantation; HCC, hepatocellular carcinoma; HR, hazard ratio.

In the subgroup analysis of patients with HCC, we observed more patients exceeding the Milan criteria in the CLT group. Thus, we performed a Cox regression analysis to adjust for HCC risk factors between the two groups, although all baseline characteristics did not reach statistical significance (*p* >0.05) (Table S3). As displayed in Fig. 2, the 5-year overall patient and graft survival rates of the IFLT group were significantly superior to those of the CLT group (82.35% *vs*. 42.03%; HR 0.249, 95% CI 0.074–0.831; *p* <0.05; and 76.47% *vs*. 42.03%; HR 0.368, 95% CI 0.127–1.060; *p* = 0.065). Moreover, the 5-year recurrence-free survival rate of IFLT recipients was also better than those of CLT recipients (76.47% *vs*. 33.33%; HR 0.324, 95% CI 0.110–0.955; *p* <0.05). Donor (type) and recipient (maximal tumor diameter, number of tumors, and tumor stages outside of the Milan and University of California San Francisco [UCSF] criteria) risk factors were covariates in the Cox regression analysis. The main reason for the higher survival rate in the IFLT group was the significantly lower incidence of tumor recurrence (5.88% *vs*. 36.23%; *p* <0.05) (Table S4). Detailed anatomical locations and time intervals of recurrences post transplantation leading to death in patients with HCC are presented in Table S5.

Risk factors associated with patient survival with HCC were explored in univariate and multivariate analyses (Table 3). In the univariate analysis, transplant techniques (IFLT *vs*. CLT), maximal tumor diameter, number of tumors, and tumor stages outside of the Milan or UCSF criteria affected the patient survival (*p* <0.05). Risk factors with *p* value <0.1 were included in the multivariate analysis. We found that the IFLT technique was an independent factor in improving long-term patient survival (HR 0.249, 95% CI 0.074–0.831; *p* <0.05), whereas extended criteria donors (HR 2.066, 95% CI 1.079–3.957; *p* <0.05) and maximal tumor diameter >30 mm (HR 3.109, 95% CI 1.294–7.470; *p* <0.05) were independent risk factors.

**Table 3:** Univariate and multivariate analyses of factors contributing to patient survival in recipients with HCC.

	Univariate analysis		Multivariate analysis∗	
	HR (95% CI)	*p* value	HR (95% CI)	*p* value
**Donor characteristics**				
Age (years)	1.011 (0.987–1.037)	0.362		
Sex				
Male	Reference	NA		
Female	0.871 (0.439–1.729)	0.693		
BMI (kg/m^2^)	0.956 (0.835–1.094)	0.513		
Cause of death				
Head trauma	Reference	NA		
Anoxia	NA	0.997		
Cerebrovascular accident	1.186 (0.651–2.159)	0.577		
Miscellaneous	1.266 (0.306–5.239)	0.745		
Donor risk index	0.799 (0.193–3.307)	0.757		
Type				
Standard criteria donor	Reference	NA		
Extended criteria donor	1.778 (0.957–3.303)	0.068	2.066 (1.079–3.957)	0.029

**Recipient characteristics**				
Age (years)	0.997 (0.967–1.028)	0.852		
Sex				
Male	Reference	NA		
Female	0.742 (0.180–3.070)	0.681		
Laboratory MELD score†	1.006 (0.972–1.041)	0.721		
Transplant techniques				
CLT	Reference	NA		
IFLT	0.232 (0.072–0.751)	0.015	0.249 (0.074–0.831)	0.024
HBV infection				
Negative	Reference	NA		
Positive	1.250 (0.594–2.629)	0.557		
Waiting time	0.997 (0.990–1.005)	0.491		
Bridging or downstaging therapy				
No	Reference	NA		
Hepatectomy	1.158 (0.358–3.744)	0.807		
LRTs	1.157 (0.623–2.148)	0.645		
Hepatectomy and LRTs	1.100 (0.433–2.797)	0.841		
LRTs and TKIs	1.300 (0.313–5.390)	0.718		
AFP (μg/L)				
≤400	Reference	NA		
>400	1.726(0.868–3.432)	0.120		
Maximal tumor diameter (mm)				
≤30	Reference	NA		
>30	3.031 (1.345–6.828)	0.007	3.109 (1.294–7.470)	0.011
Number of tumors				
≤3	Reference	NA		
>3	2.262 (1.239–4.131)	0.008	1.516 (0.702–3.273)	0.290
Partial hepatectomy				
No	Reference	NA		
Yes	1.135 (0.526–2.447)	0.747		
Milan criteria				
In	Reference	NA		
Out	2.443 (1.168–5.113)	0.018	0.430 (0.089–2.079)	0.294
UCSF criteria				
In	Reference	NA		
Out	2.963 (1.486–5.906)	0.002	3.076 (0.673–14.064)	0.147
Child–Pugh class				
A	Reference	NA		
B	0.688 (0.367–1.288)	0.242		
C	1.101 (0.511–2.375)	0.806		
BCLC stage				
0	Reference	NA		
A	0.908 (0.447–1.845)	0.790		
B	1.176 (0.613–2.257)	0.626		
C	1.308 (0.670–2.550)	0.431		
D	1.101 (0.511–2.375)	0.806		
ECOG score				
0	Reference	NA		
1	0.973 (0.383–2.474)	0.955		
2	NA	0.996		

**Immunosuppressive regimen**				
MMF				
No	Reference	NA		
Yes	0.677 (0.367–1.248)	0.211		
mTOR				
No	Reference	NA		
Yes	0.733 (0.395–1.361)	0.325		

**Operation characteristics**				
Anhepatic phase (min)	1.001 (0.984–1.018)	0.948		
Recipient operation time (min)				
≤600	Reference	NA		
>600	0.978 (0.236–4.047)	0.976		
Blood loss (ml)				
≤2,000	Reference	NA		
>2,000	0.977 (0.516–1.849)	0.943		
Intraoperative use of RBCs (ml)				
≤2,000	Reference	NA		
>2,000	1.315 (0.406–4.254)	0.648		
Intraoperative use of FFP (ml)				
≤2,000	Reference	NA		
>2,000	0.966 (0.448–2.082)	0.929		

Univariate and multivariate analyses were performed using Cox regression. Statistical significance was set at *p* <0.05.

AFP, alpha-fetoprotein; BCLC, Barcelona Clinic Liver Cancer; CLT, conventional liver transplantation; ECOG, Eastern Cooperative Oncology Group Performance Status; FFP, fresh frozen plasma; HCC, hepatocellular carcinoma; HR, hazard ratio; IFLT, ischemia-free liver transplantation; INR, international normalized ratio; LRT, locoregional therapy; MELD, model for end-stage liver disease; MMF, mycophenolate mofetil; mTOR, mammalian target of rapamycin; NA, not applicable; RBC, red blood cell; TKI, tyrosine kinase inhibitor; UCSF, University of California San Francisco; UNOS, United Network for Organ Sharing.

∗ Variables with a *p* value <0.1 in the univariate analysis were included in the multivariate analysis.

† Laboratory MELD score was calculated using the formula 3.8[Ln serum bilirubin (mg/dl)] + 11.2[Ln INR] + 9.6[Ln serum creatinine (mg/dl)] + 6.4, as described by the UNOS for liver transplant prioritization.

### Liver-related complications

Symptomatic NASs occurred exclusively in the CLT group, with no cases in the IFLT group (Table 1).^5^ Among three patients with NAS in the CLT group, one received endoscopic stenting and two required long-term use of choleretic drugs. Cholangitis also occurred only in the CLT group and required antibiotic therapy. Additional biliary complications including anastomotic strictures, bile leaks, and bile stones were comparable between the groups.

Acute rejection occurred less frequently in the IFLT group, with 7 out of 38 recipients (18.42%), compared with 39 out of 130 recipients (30.00%) in the CLT group. In addition, there were comparable infection rates between the two groups, including pneumonia, sepsis, and other types of infections (Table 1).

### Liver function tests

Results of post-transplant liver and renal biochemical blood tests are shown by year in Fig. S2. Some indicators reflecting the severity of acute IRI, including ALT, AST, and TBIL, were significantly lower in the IFLT group within 1 week postoperatively. However, these differences gradually disappeared during long-term follow-up. IFLT recipients had a long-term lower trend of ALP and GGT levels, which are related to bile duct injury, but the differences did not reach statistical significance. Other parameters, including ALT, AST, TBIL, GLU, PA, and CREA, were comparable between the groups during long-term follow-up.

## Discussion

This is the first 5-year follow-up study on the IFLT technique, demonstrating that its benefits are not limited to reducing early-onset IRI-related complications but also contribute to improving patient and graft survival.

Previous studies have shown that IFLT can reduce the incidence of IRI-related complications, including EAD, post-reperfusion syndrome, asymptomatic NAS, and a 12-month comprehensive complication index.[11], [12], [13] These studies mainly used the incidence of EAD as the primary endpoint during a 1-year follow-up. However, the latest consensus on liver machine perfusion argues against EAD as a good surrogate for long-term graft and patient survival, which are the gold standard for assessing clinical benefit.^18^ Recently, the results of 5-year follow-ups on NMP and HOPE have been reported and have shown that these techniques can improve 5-year graft survival rates compared with CLT using static cold storage in high-risk donor liver transplants including ECD-DBD, DCD, and discarded livers.[19], [20], [21], [22], [23] In the current study, we show for the first time that IFLT improves 5-year patient and graft survival in recipients of DBD liver transplants.

The survival benefit of the IFLT technique is mainly explained by significantly reduced liver-related graft loss (5.26% *vs*. 34.82%) as a consequence of PNF, HAT, and tumor recurrence. Previous studies have confirmed that IRI, as an inevitable component of liver transplantation, could lead to PNF or even patient death in severe cases.^3^ By using IFLT, graft IRI can be largely avoided.^10^ Moreover, compared with NMP preservation alone, viability tests can be done more quickly and accurately because there is no ischemia before and after *ex situ* NMP preservation during IFLT.^24^^,^^25^ Therefore, the occurrence of PNF can be avoided using IFLT. Interestingly, the incidence of HAT was reduced in the IFLT *vs*. CLT recipients, which might be explained by the use of a donor celiac artery for anastomosis and activation of fibrinolysis during *ex situ* NMP.[26], [27], [28] Therefore, IFLT can improve graft survival censored for tumor-related death and patient survival censored for tumor-related graft loss by avoiding PNF and reducing HAT.

Importantly, IRI can affect and alter the immune microenvironment of the graft, making it more susceptible to tumor recurrence.^29^ Clinical studies have shown that severe IRI is associated with a higher rate of tumor recurrence in transplant recipients with HCC.^15^^,^^30^^,^^31^ Previous studies have shown that IRI triggers cancer recurrence through CXCL10/CXCR3 signaling to mobilize regulatory T cells.^32^ Our previous experimental study has shown that IFLT can largely abrogate IRI.^10^ Moreover, the CXCR3 and cytokine–cytokine receptor interaction pathways are downregulated in the IFLT grafts. In the current study, the results of subgroup Cox regression analysis show that both patient overall survival and recurrence-free survival are improved in patients with HCC receiving IFLT after adjustment for HCC risk factors, including HCC stages. Moreover, our results show that IFLT can substantially reduce cancer recurrence rate compared with CLT, which contributes to its long-term survival benefit. Data from a national cohort study showed that less than 40% of patients with HCC met the Milan criteria at the time of transplant in China.^33^ For patients with HCC exceeding the Milan criteria, the 5-year recurrence rate exceeds 40%, compared with less than 20% for those within the Milan criteria.^34^ The high proportion of cases beyond the Milan criteria in our study likely contributed to the observed high recurrence and low survival rates in the CLT group. Notably, the CLT group had a slightly higher proportion of patients exceeding the Milan criteria, which may have exaggerated the survival difference between the groups. To address this, we performed Cox regression and multivariate analyses, adjusting for key HCC risk factors. The adjusted results continued to support the advantages of IFLT in reducing recurrence rates and improving long-term survival, particularly in high-risk patients with HCC, likely by mitigating the effects of graft IRI on cancer recurrence.

Allograft rejection is also a major cause of late graft loss.^35^ Studies have shown that IRI can trigger alloimmune responses by reactive oxygen species production, which induces damage-associated molecular patterns, leading to dendritic cell activation and the subsequent initiation of adaptive alloimmunity.^36^ Several studies have demonstrated that novel preservation techniques ameliorate the consequences of IRI with a reduced risk of acute rejection.^20^^,^^37^^,^^38^ Our experimental study has shown that redox hemostasis was maintained in IFLT grafts but not in CLT grafts.^10^ In the current study, we also documented a reduction in rejection rates among IFLT recipients.

Undoubtedly, there are some limitations to this study. First, because of the limited availability of disposable sets and perfusionists for machine perfusion, we initially designed a non-randomized trial to evaluate the safety and effectiveness of IFLT. Although donor and recipient characteristics were balanced between groups, selection bias cannot be entirely ruled out. To this end, a multivariate Cox proportional hazards regression was further performed to adjust for potential baseline differences. Second, the outcomes reported in this study were not the primary endpoints designed in the original study protocol, which limits multiple testing for numerous parameters. Hence, the analysis of these outcomes should be considered exploratory. Third, patients in this study were from a single center, and the sample size was relatively small, especially in the subgroup analysis, which may limit the generalizability of the findings. Nevertheless, our data are unique and based on an entirely novel approach that improves outcomes significantly while serving as a platform for detailed mechanistic studies. Lastly, additional procurement time and the seamless collaboration of the multiorgan procurement teams are required to ensure the procurement of other donor organs via the DBD procedure when IFLT is conducted.

In conclusion, the results of the current study demonstrate the potential long-term benefits of IFLT in improving 5-year patient and graft survival, thus serving as a basis for future multicenter randomized trials.

## Abbreviations

ALP, alkaline phosphatase; ALT, alanine aminotransferase; AST, aspartate aminotransferase; CLT, conventional liver transplantation; CREA, creatinine; DBD, donation after brain death; DCD, donation after cardiac death; EAD, early allograft dysfunction; GGT, gamma-glutamyltransferase; GLU, glucose; HAT, hepatic artery thrombosis; HCC, hepatocellular carcinoma; HOPE, hypothermic oxygenated perfusion; HR, hazard ratio; IFLT, ischemia-free liver transplantation; IRI, ischemia–reperfusion injury; NAS, non-anastomotic stricture; NMP, normothermic machine perfusion; PA, prealbumin; PNF, primary non-function; RCT, randomized controlled trial; TBIL, total bilirubin; UCSF, University of California San Francisco.

## Financial support

This study was supported through the following grants: the National Natural Science Foundation of China (81970564, 82070670, 82170663, 82370664, and 82300744), the Guangdong Provincial Key Laboratory Construction Projection on Organ Donation and Transplant Immunology (2023B1212060020), the Guangdong Provincial International Cooperation Base of Science and Technology (Organ Transplantation) (2020A0505020003), and the Science and Technology Program of Guangdong (2024B1515040011). This study was also supported by the China Organ Transplantation Development Foundation. The funders/sponsors had no role in the design and conduct of the study; collection, management, analysis, and interpretation of the data; preparation, review, or approval of the manuscript; and decision to submit the manuscript for publication.

## Authors’ contributions

Had full access to all of the data in the study and take responsibility for the integrity of the data and the accuracy of the data analysis: XH, ZG. Had access to the trial results and reviewed and approved the final version of the manuscript for publication: all authors. Served as the principal investigator for the study: XH, ZG. Contributed to study design: XH, ZG, ZJ, CH. Drafted the protocol: XH, ZG, ZJ, CH. Enrolled the patients and conducted the operations: TW, ZC, YT, QZ, YC, MC, AH, WJ, YM, DW, XZ. Collected the data: JZ, NZ, SL. Analyzed the data: ZJ, JZ, NZ, SL, YD, YL. Interpreted the data: XH, ZG, ZJ, JZ, NZ, SL, YD, YL. Drafted the manuscript: XH, ZG, ZJ. Participated in paper writing and editing: ZJ, AS, SGT.

## Data availability statement

Data can be made available to researchers upon reasonable request.

## Conflicts of interest

The authors of this study declare that they do not have any conflict of interest.

Please refer to the accompanying ICMJE disclosure forms for further details.
