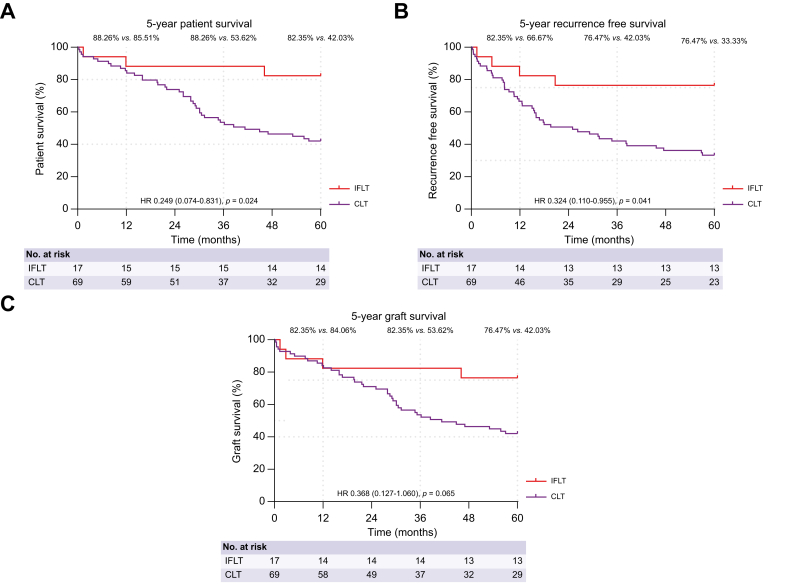# Erratum to ‘Ischemia-free liver transplantation improves long-term outcomes in a 5-year follow-up study’ (JHEP Reports 7 [2025] 101393)

**DOI:** 10.1016/j.jhepr.2026.101751

**Published:** 2026-02-17

**Authors:** Zehua Jia, Jiaxing Zhu, Jiayi Zhang, Jian Zhang, Changjun Huang, Niancun Zhang, Songming Li, Yuqi Dong, Yao Liu, Ping Zeng, Tielong Wang, Zhitao Chen, Yunhua Tang, Qiang Zhao, Maogen Chen, Yinghua Chen, Anbin Hu, Weiqiang Ju, Yi Ma, Dongping Wang, Xiaofeng Zhu, Andrea Schlegel, Tullius G. Stefan, Xiaoshun He, Zhiyong Guo

**Affiliations:** 1 Organ Transplant Center, The First Affiliated Hospital, Sun Yat-sen University, Guangzhou, China; 2 Guangdong Provincial Key Laboratory of Organ Medicine, Guangzhou, China; 3 Guangdong Provincial International Cooperation Base of Science and Technology (Organ Transplantation), Guangzhou, China; 4 State Key Laboratory of Ophthalmology, Zhongshan Ophthalmic Center, Sun Yat-sen University, Guangzhou, China; 5 Transplantation Center, Digestive Disease and Surgery Institute and Department of Immunology, Lerner Research Institute, Cleveland Clinic, Cleveland, OH, USA; 6 Division of Transplant Surgery, Brigham and Women's Hospital, Harvard Medical School, Boston 02115, MA, USA; 7 NHC Key Laboratory of Assisted Circulation, Sun Yat-sen University, Guangzhou, China

It has come to our attention that errors were present in Figures 1 and 2 of the published version of our manuscript. During the production process, the x-axes of the survival curves were incorrectly labeled as “Time (days)” instead of “Time (months).” This error has been corrected in the online version, and the corrected figures are provided below. We apologize for any inconvenience caused.

**Fig. 1:**
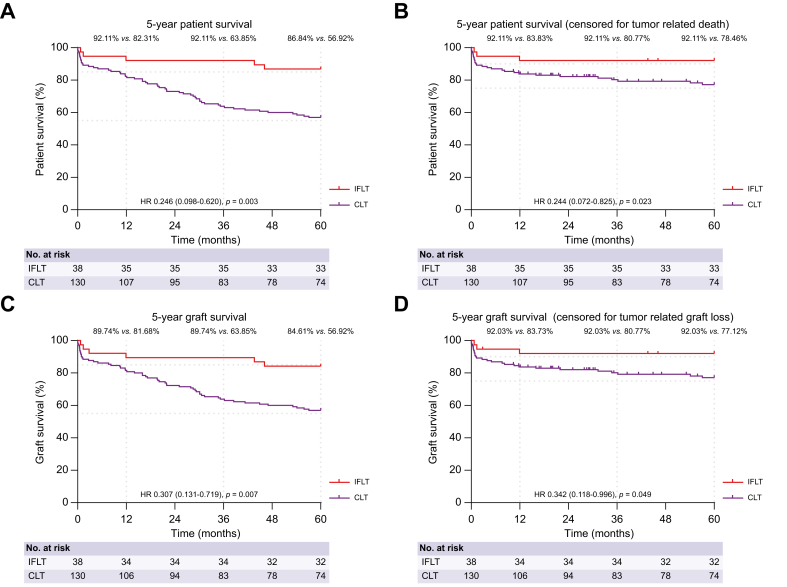

**Fig. 2:**