# Supplementary material for: Ischemia-free liver transplantation improves long-term outcomes in a 5-year follow-up study
Source: JHEP Rep. 2025 Mar 12;7(7):101393. doi: 10.1016/j.jhepr.2025.101393 (PMC12167472; doi:10.1016/j.jhepr.2025.101393)
Supplement: Multimedia component 1 [file mmc1.pdf]

Ischemia-free liver transplantation improves long-term outcomes in a 5-year follow-up study

Zehua Jia, Jiaxing Zhu, Jiayi Zhang, Jian Zhang, Changjun Huang, Niancun Zhang, Songming Li, Yuqi Dong, Yao Liu, Ping Zeng, Tielong Wang, Zhitao Chen, Yunhua Tang, Qiang Zhao, Maogen Chen, Yinghua Chen, Anbin Hu, Weiqiang Ju, Yi Ma, Dongping Wang, Xiaofeng Zhu, Andrea Schlegel, Tullius G. Stefan, Xiaoshun He, Zhiyong Guo

Table of contents

Supplementary methods……………………………………………………………….2

Supplementary figures……………………………………………………………..…...3

Supplementary tables…………………………………………………………….......5

Supplementary methods

Post-transplant immunosuppression protocol

Standardized post-transplant immunosuppressive treatment was provided for both groups. The immunosuppression (IS) therapy was divided into induction and maintenance phases as follows:

- Induction phase: Basiliximab (20mg) was administered intravenously during the operation and at post-operative day (POD) 4.
- Maintenance phase: The maintenance therapy began at POD 4. The mainstay of maintenance therapy is the combined use of tacrolimus and mycophenolic acid or sirolimus. The initial dose of tacrolimus was 0.04 mg/kg/d, and the target trough level was 8-10 ng/ml within the first three months, and 6-8 ng/ml thereafter.

Post-transplant tumor recurrence surveillance protocol for patients with liver cancer

Standardized post-transplant tumor recurrence surveillance was implemented. This included regular monitoring of alpha-fetoprotein (AFP) levels and imaging studies:

- AFP Monitoring: Conducted monthly during the first year and every 2-3 months from the second to fifth year post-transplantation.
- Imaging (CT/MRI/ultrasound): Performed every 6 months during the first year and annually from the second to fifth year post-transplantation.

Additionally, AFP levels and imaging studies were promptly conducted for any patients presenting with abnormal clinical signs or test results suggestive of tumor recurrence. This consistent surveillance ensured the reliable detection of tumor recurrence in both groups.

Supplementary figures

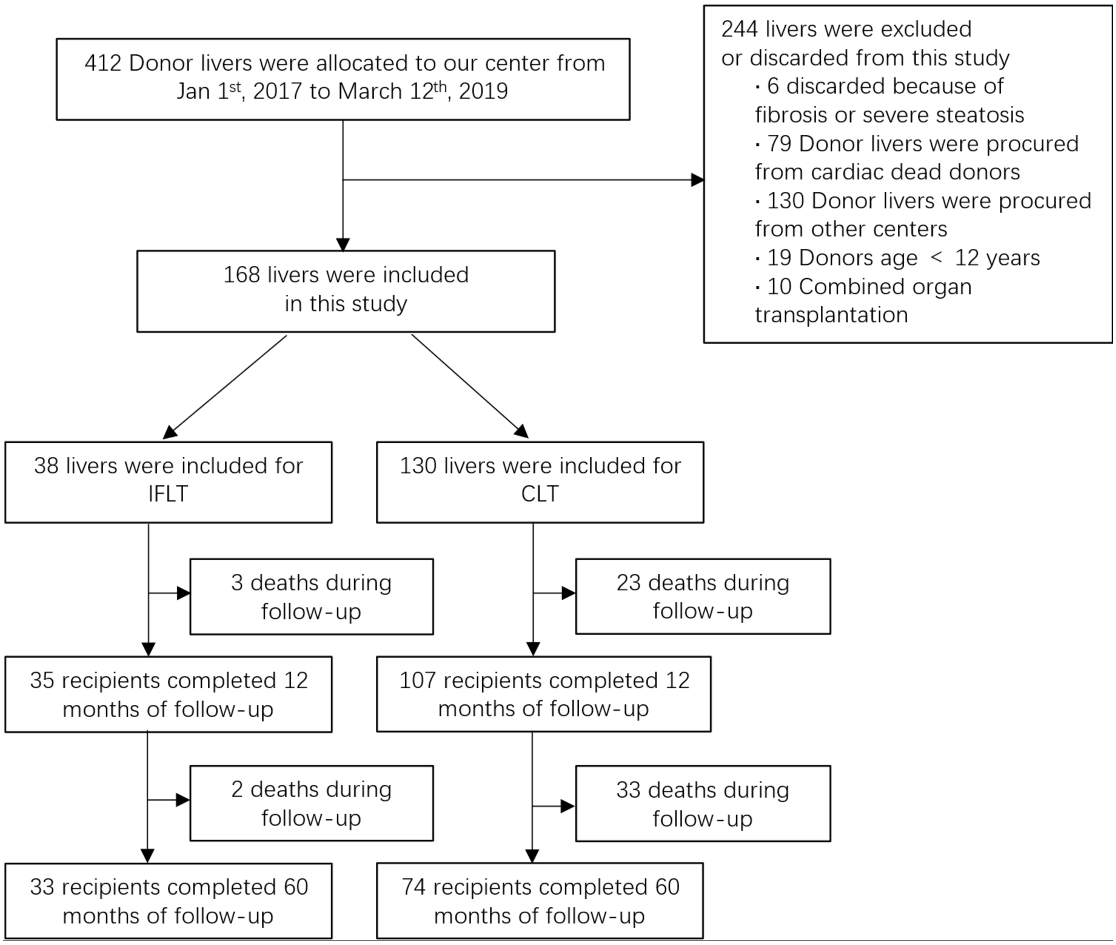

Fig. S1. Flow diagram of patient screening, selection and follow-up.

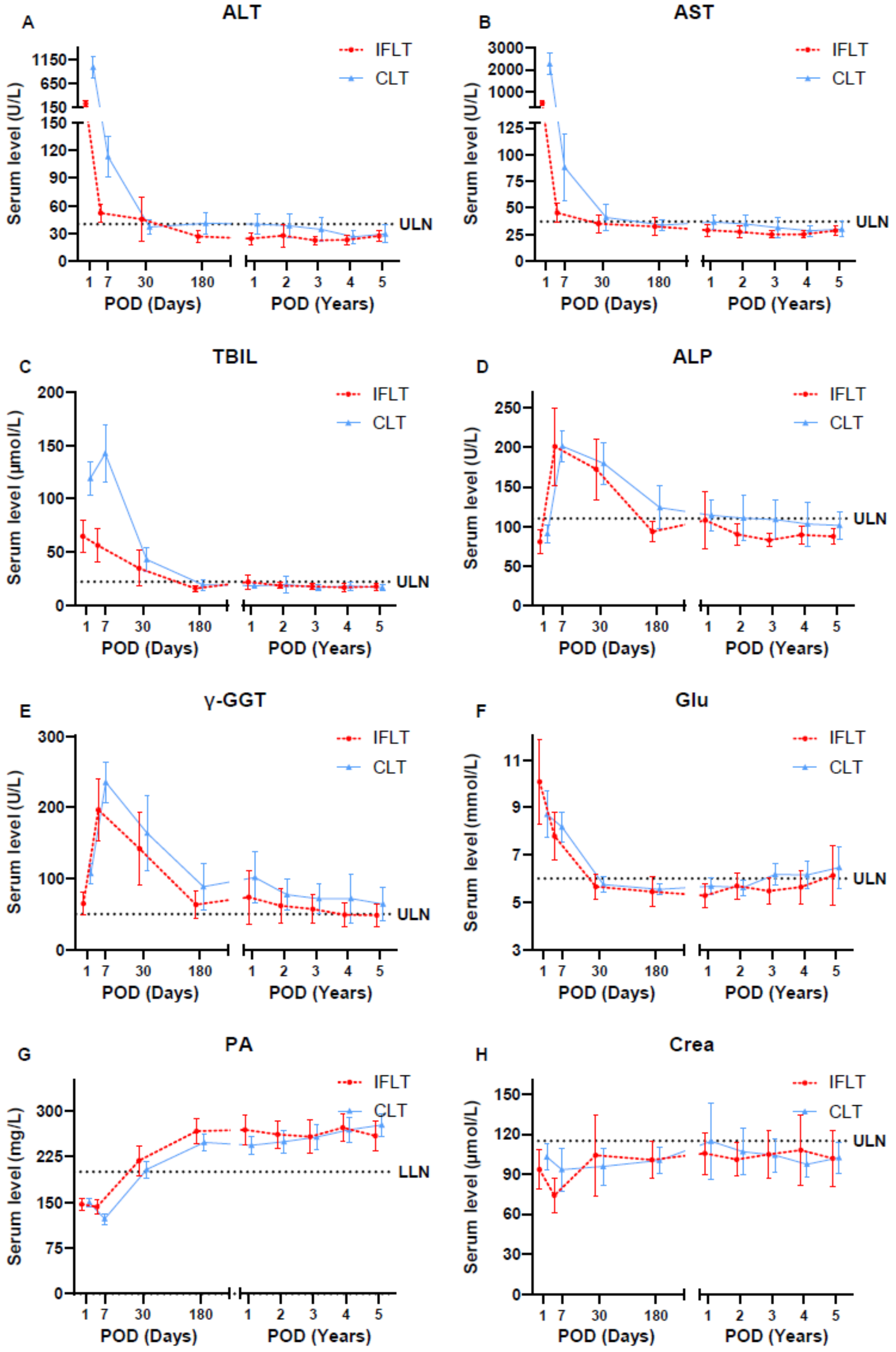

Fig. S2. Liver function tests after liver transplantation.

Continuous variables are presented as mean and 95%CI (Confidence Interval). IFLT, ischemia-free liver transplantation; CLT, conventional liver transplantation; ULN, upper limit of normal; LLN, lower limit of normal; PA, prealbumin; TBIL, total bilirubin; Glu, glucose; Crea, creatinine; γGGT, gamma-glutamyl transferase; LDH, lactate dehydrogenase; ALP, alkaline phosphatase; ALT, alanine aminotransferase; AST, aspartate aminotransferase.

Supplementary tables

Table S1. Baseline demographic and clinical characteristics of donors and recipients.

	IFLT (n=38)	CLT (n=130)	*p* value
**Donor characteristics**			
Age, mean (SD), years	36 (14)	37 (12)	0.597
Male, No. (%)	31 (81.58)	94 (72.31)	0.347
BMI, mean (SD)	22.27 (2.16)	22.47 (2.45)	0.648
Cause of death, No. (%)			0.161
Head trauma	21 (55.26)	55 (42.31)	
Anoxia	3 (7.89)	9 (6.92)	
Cerebrovascular accident	11 (28.95)	62 (47.69)	
Miscellaneous†	3 (7.89)	4 (3.08)	
Donor risk index, mean (SD)	1.34 (0.24)	1.36 (0.21)	0.656
Extended criteria donor, No. (%)‡	12 (31.58)	29 (22.31)	0.339
Macrosteatosis, No. (%)			0.643
No/Mild (<30%)	36 (94.74)	125 (96.15)	
Moderate (30%-60%)	1 (2.63)	4 (3.08)	
Severe (>60%)	1 (2.63)	1 (0.77)	
**Recipient characteristics**			
Age, mean (SD), years	51 (11)	50 (10)	0.741
Male, No. (%)	34 (89.47)	120 (92.31)	0.824
Laboratory MELD score, mean (SD) §	23.94 (6.69)	24.58 (8.62)	0.670
HBV positive, No. (%)	32 (84.21)	110 (84.62)	1.000
Waiting time, mean (SD), days	30 (49)	31 (60)	0.885
Principal diagnosis, No. (%)			0.182
HCC	17 (44.74)	69 (53.08)	
Decompensate cirrhosis	16 (42.11)	32 (24.62)	
Liver failure	4 (10.53)	25 (19.23)	
Miscellaneous¶	1 (2.63)	4 (3.08)	
**Operation characteristics**			
Liver retrieval time, median (IQR), min	204 (190-220)	45 (40-59)	<0.001
NMP duration time, median (IQR), min	240 (160-360)	NA	NA
Cold ischemia time, median (IQR), min	NA	369 (329-450)	NA
Anhepatic time, mean (SD), min	52 (15)	53 (20)	0.706
Recipient operation time > 600 min, No. (%)	0	9 (6.92)	0.208
Blood loss > 2000 ml, No. (%)	16 (42.11)	53 (40.77)	1.000
Intraoperative use of RBCs > 2000 ml, No. (%)	4 (10.53)	27 (20.77)	0.232
Intraoperative use of FFP > 2000 ml, No. (%)	10 (26.32)	37 (28.46)	0.957

* Continuous variables were compared using t-test or the Mann-Whitney U test, while categorical variables were analyzed using the chi-square test and Fisher's exact test. Statistical significance was set at $p < 0.05$. IFLT, ischemia-free liver transplantation; CLT, conventional liver transplantation; BMI, body mass index; MELD, model for end-stage liver disease; HBV, hepatitis B virus; HCC, hepatocellular carcinoma; NMP, normothermic machine perfusion; RBC, red blood cell; FFP, fresh frozen plasma.

† Miscellaneous includes bacterial encephalitis, viral encephalitis, organophosphorus poisoning.

‡ Extended criteria donor (ECD) was defined as meeting at least one of the following criteria: (1) donor age >60 years; (2) hypernatremia (serum Na^+ >165 mmol/L); (3) >30% macrovesicular steatosis by biopsy; (4) donor serum aspartate aminotransferase (AST) or alanine amino- transferase (ALT) >1,000 IU/L or total bilirubin (Tbil) >3 mg/dL before procurement; (5) or cold ischemia time (CIT) ⩾12 hours.

§ Laboratory MELD score was calculated using the formula: 3.8 [Ln serum bilirubin (mg/dL)] + 11.2[Ln INR] + 9.6[Ln serum creatinine (mg/dL)] + 6.4, as described by the United Network for Organ Sharing (UNOS) for liver transplant prioritization.

¶ Miscellaneous includes Budd-Chiari syndrome, cholangiocarcinoma, hepatic mucinous cystic neoplasm, hepatic epithelioid hemangioendothelioma.

Table S2. Cox regression analysis of 5-year patient and graft survival.

Outcomes	Hazard ratio (95% CI)	*p* value
5-year patient survival		
Unadjusted	0.252 (0.101-0.628)	0.003
Adjusted*	0.246 (0.098-0.620)	0.003
5-year patient survival (censored for tumor related death)		
Unadjusted	0.324 (0.098-1.070)	0.064
Adjusted*	0.244 (0.072-0.825)	0.023
5-year graft survival		
Unadjusted	0.308 (0.133-0.716)	0.006
Adjusted*	0.307 (0.131-0.719)	0.007
5-year patient graft (censored for tumor related graft loss)		
Unadjusted	0.422 (0.148-1.200)	0.110
Adjusted*	0.342 (0.118-0.996)	0.049

* The hazard ratio (HR) and *p* value were calculated using the Cox regression adjusted for donor (age and type) and recipient (HCC prior to transplantation) risk factors. Statistical significance was set at $p < 0.05$.

Table S3. Donor, recipient and operation characteristics in patients with HCC prior to transplantation.*

	IFLT (n=17)	CLT (n=69)	*p* value
**Donor characteristics**			
Age, mean (SD), years	34 (12)	38 (12)	0.197
Male, No. (%)	13 (76.47)	49 (71.01)	0.883
BMI, mean (SD)	22.22 (2.05)	22.63 (2.29)	0.501
Cause of death, No. (%)			0.182
Head trauma	10 (58.82)	30 (43.48)	
Anoxia	1 (5.88)	4 (5.80)	
Cerebrovascular accident	4 (23.53)	33 (47.83)	
Miscellaneous†	2 (11.76)	2 (2.90)	
Donor risk index, mean (SD)	1.29 (0.17)	1.36 (0.21)	0.239
Extended criteria donor, No. (%)	7 (41.18)	19 (27.54)	0.423
Macrosteatosis, No. (%)			0.643
No/Mild (<30%)	15 (88.24)	66 (95.65)	
Moderate (30%-60%)	1 (5.88)	3 (4.35)	
Severe (>60%)	1 (5.88)	0 (0)	
**Recipient characteristics**			
Age, mean (SD), years	51 (11)	51 (10)	0.894
Male, No. (%)	16 (94.12)	65 (94.20)	1.000
HBV positive, No. (%)	15 (88.2)	66 (95.7)	0.554
Waiting time, mean (SD), days	39 (67)	27 (38)	0.347
Bridging or downstaging therapy			0.159
No	11 (64.71)	28 (40.58)	
Hepatectomy	1 (5.88)	5 (7.25)	
LRTs	2 (11.76)	27 (39.13)	
Hepatectomy and LRTs	3 (17.65)	6 (8.70)	
LRTs and TKIs	0 (0)	3 (4.35)	
Laboratory MELD score, mean (SD)‡	20.47 (5.02)	24.14 (8.98)	0.109
AFP >400 μg/L, No. (%)	3 (17.65)	14 (20.29)	1.000
Maximal tumor diameter > 30 mm, No. (%)	9 (52.94)	49 (71.01)	0.256
Number of tumors > 3, No. (%)	4 (23.53)	28 (40.58)	0.306
In Milan criteria, No. (%)	8 (47.06)	21 (30.43)	0.311
In UCSF criteria, No. (%)	10 (58.82)	27 (39.13)	0.232
Child-Pugh class, No. (%)			0.242
A	4 (23.53)	30 (43.48)	
B	8 (47.06)	28 (40.58)	
C	5 (29.41)	11 (15.94)	
BCLC stage, No. (%)			0.753
0	1 (5.88)	4 (5.80)	
A	3 (17.65)	17 (24.64)	
B	5 (29.41)	20 (28.99)	
C	3 (17.65)	17 (24.64)	
D	5 (29.41)	11 (15.94)	
ECOG score, No. (%)			0.097
0	13 (76.47)	61 (88.41)	
1	3 (17.65)	8 (11.59)	
2	1 (5.88)	0	
Immunosuppressive regimen, No. (%)			
CNIs	17 (100.00)	69 (100.00)	1.000
MMF	10 (58.82)	46 (66.67)	0.746
mTOR	5 (29.41)	29 (42.03)	0.499

**Operation characteristics**			
Anhepatic time, mean (SD), min	48 (13)	51 (19)	0.547
Recipient operation time > 600 min, No. (%)	0	4 (5.80)	0.709
Blood loss > 2000 ml, No. (%)	6 (35.29)	22 (31.88)	1.000
Intraoperative use of RBCs > 2000 ml, No. (%)	1 (5.88)	4 (5.80)	1.000
Intraoperative use of FFP > 2000 ml, No. (%)	3 (17.65)	13 (18.84)	1.000

* Continuous variables were compared using t-test or the Mann-Whitney U test, while categorical variables were analyzed using the chi-square test and Fisher's exact test. Statistical significance was set at $p < 0.05$. HCC, hepatocellular carcinoma; IFLT, ischemia-free liver transplantation; CLT, conventional liver transplantation; BMI, body mass index; MELD, model for end-stage liver disease; HBV, hepatitis B virus; TKI, tyrosine kinase inhibitors; LRT, locoregional therapy; AFP, alpha-fetoprotein; UCSF, the University of California, San Francisco criteria; BCLC, the Barcelona Clinic Liver Cancer staging classification; ECOG, Eastern Cooperative Oncology Group Performance Status; CNI, calcineurin inhibitor; MMF, mycophenolate mofetil; mTOR, mammalian target of rapamycin; RBC, red blood cell; FFP, fresh frozen plasma.

† Miscellaneous includes bacterial encephalitis, viral encephalitis, organophosphorus poisoning.

‡ Laboratory MELD score was calculated using the formula: 3.8 [Ln serum bilirubin (mg/dL)] + 11.2[Ln INR] + 9.6[Ln serum creatinine (mg/dL)] + 6.4, as described by the United Network for Organ Sharing (UNOS) for liver transplant prioritization.

Table S4. Causes of death in patients with HCC prior to transplantation.*

Causes of patient death	IFLT (n=17)	CLT (n=69)	*p* value
Tumor recurrence	1(5.88)	25(36.23)	0.02
Hepatic artery thrombosis	0	3(4.35)	1.00
Acute pulmonary embolism	1(5.88)	0	0.20
Primary non-function	0	1(1.45)	1.00
Sepsis/infection	1(5.88)	6(8.70)	1.00
Rejection	0	1(1.45)	1.00
Miscellaneous†	0	4(5.80)	0.60

* The chi-square test or Fisher's exact test was used to calculated *p* values, with statistical significance defined as $p < 0.05$. Data are presented as n (%). IFLT, ischemia-free liver transplantation; CLT, conventional liver transplantation.

† Miscellaneous includes one case of vehicle accident, one hip fracture and head trauma, one suicide in the conventional liver transplantation group only. The cause of death of one patient in the conventional liver transplantation group is unclear as the patient's family refused to provide further information.

Table S5. Detailed anatomical locations and time interval of recurrences post-transplantation leading to death in patients with HCC.*

Number ID	Anatomical locations of recurrences	Time interval of recurrence after transplantation (days)
IFLT-24	liver and lung	621
CLT-8	bile duct	1370
CLT-11	liver	343
CLT-21	liver and lung	376
CLT-23	liver, lymph nodes, retroperitoneal region, pancreas and bone	451
CLT-24	liver and lung	949
CLT-27	lung and bone	54
CLT-28	liver and lung	377
CLT-32	liver	481
CLT-37	lung	788
CLT-43	lung	245
CLT-46	lung	1153
CLT-50	liver	533
CLT-51	Greater omentum and peritoneum	161
CLT-79	lung	589
CLT-80	liver	280
CLT-83	lung	114
CLT-97	liver	14
CLT-105	lung	246
CLT-108	liver and lung	463
CLT-111	liver	44
CLT-112	lung and adrenal gland	876
CLT-113	lung	237
CLT-116	liver	155
CLT-119	liver	67
CLT-124	lung	1147

* IFLT, ischemia-free liver transplantation; CLT, conventional liver transplantation.
