# Supplementary material for: Ischemia-free liver transplantation improves long-term outcomes in a 5-year follow-up study
Source: JHEP Rep. 2025 Mar 12;7(7):101393. doi: 10.1016/j.jhepr.2025.101393 (PMC12167472; doi:10.1016/j.jhepr.2025.101393)
Supplement: Multimedia component 4 [file mmc4.pdf]

Ischemia-free liver transplantation improves long-term outcomes in a 5-year follow-up study

Authors

Zehua Jia, Jiaxing Zhu, Jiayi Zhang, …, Tullius G. Stefan, Xiaoshun He, Zhiyong Guo

Correspondence

rockyucsf1981@126.com (Z. Guo), gdtrc@163.com (X. He).

Graphical abstract

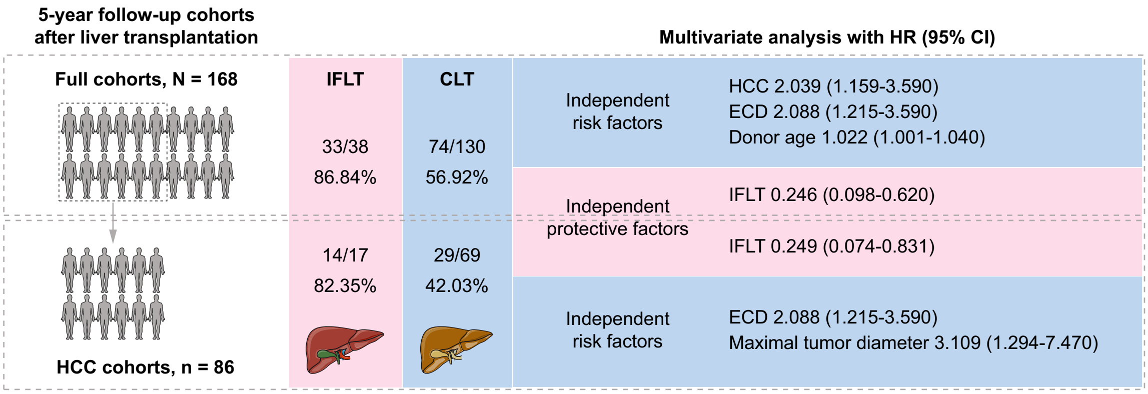

Highlights:

- This is the first 5-year follow-up study to document critical components of long-term outcomes on the IFLT technique.
- Compared with conventional liver transplantation, IFLT significantly improved both 5-year patient and graft survival rates.
- IFLT reduces various long-term complications, including tumor recurrence.

Impact and implications:

Ischemia-free liver transplantation (IFLT) has emerged as a new approach designed to avoid IRI throughout all episodes of the transplant procedure. It has been confirmed that the use of IFLT can substantially reduce early-onset graft IRI-related complications. In this first 5-year follow-up study on the IFLT technique, we demonstrate that, compared with conventional liver transplantation, IFLT can potentially improve long-term patient and graft survival by reducing cancer recurrence. This new technique has the potential to change current clinical practice, particularly in the use of marginal grafts and in patients with HCC.

https://doi.org/10.1016/j.jhepr.2025.101393
© 2025 The Authors. Published by Elsevier B.V. on behalf of European Association for the Study of the Liver (EASL). This is an open access article under the CC BY license (http://creativecommons.org/licenses/by/4.0/).

Ischemia-free liver transplantation improves long-term outcomes in a 5-year follow-up study

Zehua Jia[1,2,3,†], **Jiaxing Zhu**[1,†], **Jiayi Zhang**[1,2,3,†], Jian Zhang[4], Changjun Huang[1,2,3], Niancun Zhang[1], Songming Li[1], Yuqi Dong[1,2,3], Yao Liu[1,2,3], Ping Zeng[1,2,3], Tielong Wang[1,2,3], Zhitao Chen[1,2,3], Yunhua Tang[1,2,3], Qiang Zhao[1,2,3], Maogen Chen[1,2,3], Yinghua Chen[1,2,3], Anbin Hu[1,2,3], Weiqiang Ju[1,2,3], Yi Ma[1,2,3], Dongping Wang[1,2,3], Xiaofeng Zhu[1,2,3], Andrea Schlegel[5], Tullius G. Stefan[6], Xiaoshun He[1,2,3,*], Zhiyong Guo[1,2,3,7,*]

JHEP Reports **2025**. vol. 7 | 1–10

Check for updates

Background & Aims: Ischemia-free liver transplantation (IFLT) is a novel technique designed to avoid ischemia–reperfusion injury (IRI). Here, we report the first detailed 5-year follow-up outcomes.

Methods: We conducted a cohort study comparing long-term outcomes between IFLT and conventional liver transplantation (CLT) recipients of livers donated after brain death (DBD). The primary objective was to evaluate 5-year patient and graft survival. Additional endpoints included graft loss, biliary complications, rejection, infections, and liver-related laboratory tests. Subgroup analysis was performed to validate the generalizability of the results in patients with pre-transplant hepatocellular carcinoma (HCC).

Results: A total of 168 patients were enrolled, with 38 patients in the IFLT group and 130 patients in the CLT group. Five-year patient survival (86.84% *vs*. 56.92%; hazard ratio [HR] 0.246, 95% confidence interval [CI] 0.098–0.620; *p* <0.01) and graft survival (84.61% *vs*. 56.92%; HR 0.307, 95% CI 0.131–0.719; *p* <0.01) rates were significantly improved in the IFLT group compared with the CLT group. In the multivariate analysis, IFLT emerged as an independent protective factor for 5-year patient survival (HR 0.246, 95% CI 0.098–0.620; *p* <0.01). Conversely, HCC before transplantation (HR 2.039, 95% CI 1.159–3.590; *p* <0.05), donor age (HR 1.022, 95% CI 1.001–1.040; *p* <0.05), and extended criteria donor (HR 2.088, 95% CI 1.215–3.590; *p* <0.01) were identified as independent risk factors for impaired 5-year patient survival. In patients with pre-transplant HCC, the 5-year overall survival rate of the IFLT group was also significantly higher than that of the CLT group after adjustment for HCC risk factors (82.35% *vs*. 42.03%; HR 0.249, 95% CI 0.074–0.831; *p* <0.05).

Conclusions: Long-term (5-year) follow-up data demonstrate that the use of IFLT potentially improves both patient and graft survival, when compared with CLT, in transplantation of brain-dead donor livers.

Clinical Trials registration: chictr.org (ChiCTR-OPN-17012090).

© 2025 The Authors. Published by Elsevier B.V. on behalf of European Association for the Study of the Liver (EASL). This is an open access article under the CC BY license (http://creativecommons.org/licenses/by/4.0/).

Introduction

Liver transplantation represents the standard of care for end-stage liver diseases.[1] Conventional liver transplantation (CLT) techniques inevitably involve ischemic periods during organ procurement, preservation, and implantation, which significantly impact short-term and long-term transplant outcomes as a consequence of ischemia–reperfusion injury (IRI).[2,3]

To mitigate the detrimental effects of IRI, various *ex situ* machine perfusion technologies, including hypothermic machine perfusion, hypothermic oxygenated perfusion (HOPE), normothermic machine perfusion (NMP), and subnormothermic machine perfusion (SNMP), have been used in liver transplantation.[4] Recent randomized controlled trials (RCTs) have demonstrated that HOPE can reduce the incidence of non-anastomotic stricture (NAS)[5] and >IIIb complications in transplantation of livers donated after circulatory death.[6] In addition, the results of RCTs have shown that NMP can ameliorate the incidence of early allograft dysfunction (EAD).[7,8] However, the grafts still experienced ischemic injury before and after *ex situ* machine perfusion, making IRI unavoidable under these conditions.

Ischemia-free liver transplantation (IFLT) has emerged as a new approach designed to avoid IRI by maintaining a continuous oxygenated blood supply throughout all episodes of the transplant procedure.[9] Our comprehensive pathological, transcriptomic, and metabolomic analyses have demonstrated that IFLT can largely abrogate graft IRI.[10] The results of the first-in-human trial have shown that the use of IFLT can substantially

* Corresponding authors. Address: Organ Transplant Center, The First Affiliated Hospital, Sun Yat-sen University, NO.58 Zhongshan Er Road, Guangzhou 510080, China. Tel.: 86-20-87306082; Fax: 86-20-8730608.
E-mail addresses: rockyucsf1981@126.com (Z. Guo), gdtrc@163.com (X. He).
† These authors contributed equally to this work as co-first authors.
https://doi.org/10.1016/j.jhepr.2025.101393

reduce graft IRI-related complications.[11] Subsequent RCTs and retrospective studies further confirmed the safety and efficacy of IFLT.[12–15] However, because of the short follow-up duration and limited sample sizes, evidence of the long-term benefits of IFLT is still lacking, and no 5-year follow-up data have been reported.

In the current study, we prospectively document critical components of long-term outcomes over a 5-year follow-up period in the same cohorts from our first-in-human IFLT trial.[11]

Patients and methods

Study, setting, and ethics

Our group conducted the first-in-human trial to assess the feasibility and safety of IFLT at The First Affiliated Hospital of Sun Yat-sen University between January 2017 and March 2019 (chictr.org: ChiCTR-OPN-17012090). Intraoperative and post-transplant care was performed according to standard protocols applied at our center. This was an investigator-initiated, single-center, prospective trial, and both cohorts were followed up for 5 years in this study. The protocol was approved by the Ethical Committee of The First Affiliated Hospital of Sun Yat-sen University, and all patients provided written informed consent.

Participants

All patients receiving liver transplantation and fulfilling eligibility criteria during the study period were enrolled in this trial. All livers were procured from brain-dead donors and allocated by the China Organ Transplant Response System (COTRS) based on the emergency of diseases and waiting time.[16] Allocation was non-randomized, and patients received IFLT based on the availability of NMP device disposables and perfusionists. Detailed eligibility criteria included the following: donation after brain death (DBD) donors aged >12 years whose organs were procured at our hospital were eligible for inclusion. Donation after cardiac death (DCD) donors were excluded. Adult recipients (>18 years) undergoing first liver-only transplantation in our hospital were included in the trial. Patients were excluded if they underwent combined organ transplantation, multi-visceral transplantation, split liver transplantation, or ABO-incompatible liver transplantation. All enrolled patients were informed of the procedural risks and provided written consent to receive IFLT.

Trial interventions

In the IFLT group, donor livers underwent ischemia-free procurement under *in situ* NMP using the Liver Assist device (Organ Assist, Groningen, Netherlands); livers were preserved and assessed for viability under *ex situ* NMP. After the recipient's hepatectomy, ischemia-free liver implantation (standard bicaval or piggyback technique) was performed under *in situ* NMP. Thus, all livers were procured, preserved, and implanted without interruption of normothermic, oxygenated blood supply.

In the CLT group, donor livers were procured using a standard *in situ* cold flushing procedure and preserved in 0–4 °C University of Wisconsin (UW) solution. After the removal of diseased livers, the donor livers were subsequently transplanted using the standard bicaval or piggyback technique.

Further details concerning study design, sample size calculation, surgical procedures, NMP protocols, and standards of perioperative care are described in detail in the original trial report and protocol.[11]

Follow-up and outcomes

All patients enrolled in the original trial were followed up for 5 years. The primary objective of this study was to document 5-year overall patient survival and graft survival. Other endpoints included 5-year patient survival censored for tumor-related deaths, long-term infections, acute and chronic rejection, biliary complications,[17] re-transplantation, and detailed causes of graft loss and patient death. In addition, we compared long-term liver-related laboratory test results between IFLT and CLT recipients, including alanine aminotransferase (ALT), aspartate aminotransferase (AST), total bilirubin (TBIL), alkaline phosphatase (ALP), gamma-glutamyltransferase (GGT), glucose (GLU), prealbumin (PA), and creatinine (CREA).

Statistical analysis

Continuous variables were compared using the *t* test or the Mann–Whitney *U* test, whereas categorical variables were analyzed using the Chi-square test and Fisher's exact test. Time-to-event outcomes are presented as Kaplan–Meier curves with hazard ratios (HRs) and *p* values calculated using multivariate Cox proportional hazards regression models. Baseline variables of donors and recipients that showed a univariate relationship with patient death ($p < 0.1$) were entered into multivariate Cox proportional hazards regression models as covariates. All statistical analyses were based on two-sided tests, and results are reported with 95% confidence intervals (CIs). The significance level for the *p* value was set at 0.05. Statistical analysis was conducted using R 4.3.2 (R Foundation for Statistical Computing, Vienna, Austria).

Results

Patient characteristics

Of 412 donor livers allocated to our center from January 1, 2017, to March 12, 2019, a total of 168 patients (38 in the IFLT group and 130 in the CLT group) were included in this study. All patients completed a 5-year follow-up without any loss or withdrawal. Twenty-three patients in the CLT group and three patients in the IFLT group had died within 1 year. By the end of the observation period (year 5), an additional 33 patient deaths occurred in the CLT group and two patient deaths in the IFLT group (Fig. S1). The baseline characteristics of donors, recipients, and surgeries are detailed in Table S1 and the initial trial report,[11] which were balanced between the IFLT and CLT groups.

Five-year patient survival

The overall patient survival between the IFLT and CLT groups at 1, 3, and 5 years was 92.11% *vs.* 82.31%, 92.11% *vs.* 63.85%, and 86.84% *vs.* 56.92%, respectively (Fig. 1A). The 5-year overall survival rate was significantly higher in the IFLT group than in the CLT group after adjustment for donor (age and type) and recipient (hepatocellular carcinoma [HCC] before transplantation) risk factors (HR 0.246, 95% CI 0.098–0.620; $p < 0.01$). The unadjusted result remained essentially the same (HR 0.252, 95% CI 0.101–0.628; $p < 0.01$) (Table S2). Notably,

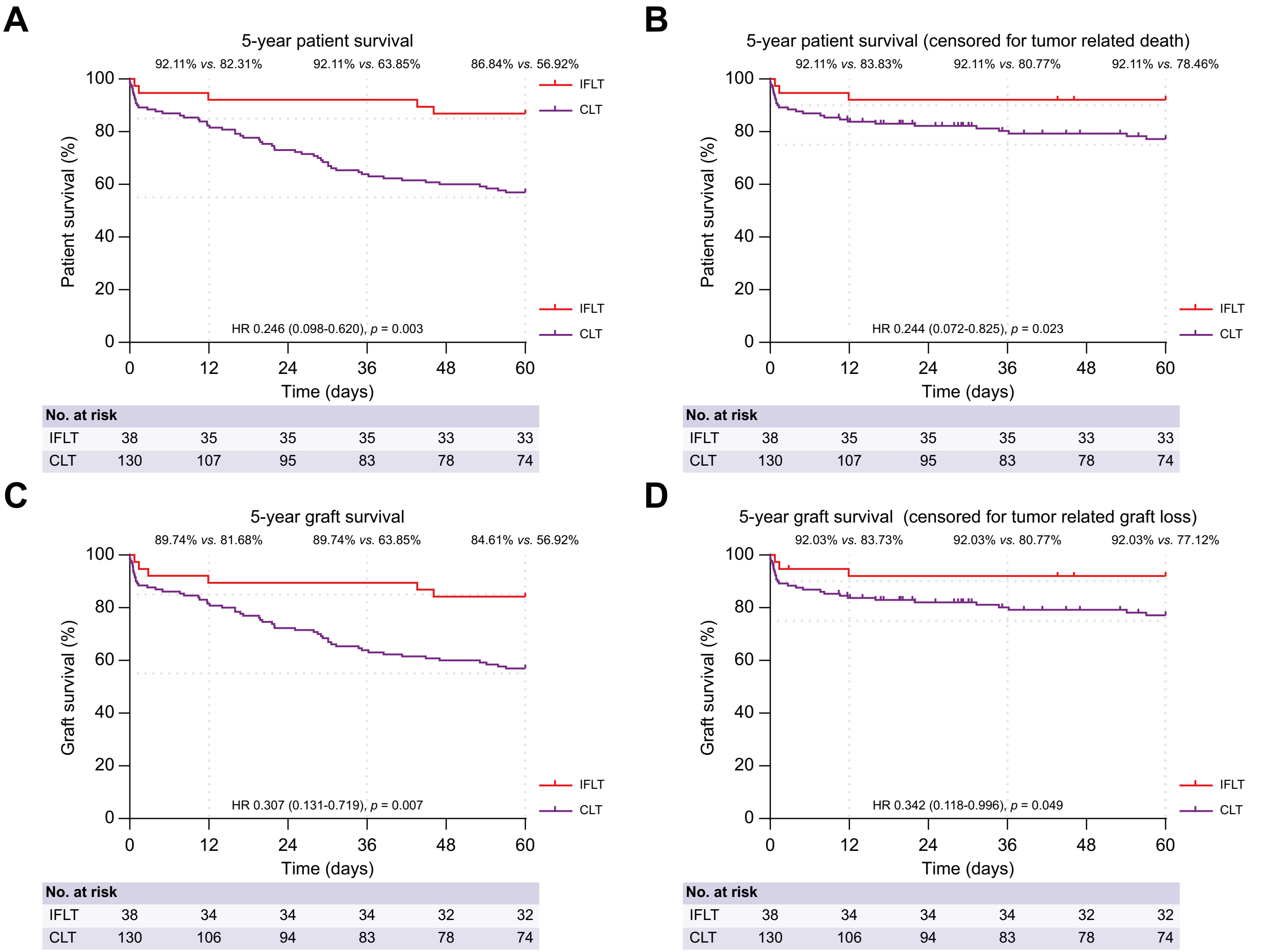

Fig. 1. Five-year patient and graft survival in the IFLT and CLT groups. Time-to-event Kaplan–Meier curves for (A) 5-year patient survival, (B) patient survival censored for tumor-related death, (C) graft survival, and (D) graft survival censored for tumor-related graft loss after liver transplantation. The HR and *p* value in (A)–(D) were calculated using the Cox regression adjusted for donor (age and type) and recipient (HCC before transplantation) risk factors. Statistical significance was set at p <0.05. CLT, conventional liver transplantation; IFLT, ischemia-free liver transplantation; HR, hazard ratio.

there was a lower incidence of graft failure (0% *vs*. 8.46%; p = 0.138) and tumor recurrence (2.63% *vs*. 21.54%; p <0.01) in the IFLT group, representing the main explanation for superior patient survival. The incidence of infections, new-onset tumors, and other unforeseen events leading to patient death were comparable between the two groups (Table 1). In addition, the 5-year survival rate censored for tumor-related death was still significantly higher in the IFLT than in the CLT group (92.11% *vs*. 78.46%; HR 0.244, 95% CI 0.072–0.825; p <0.05) (Fig. 1B).

Five-year graft survival

The overall graft survival comparing IFLT and CLT recipients at 1, 3, and 5 years was 89.74% *vs*. 81.68%, 89.74% *vs*. 63.85%, and 84.61% *vs*. 56.92%, respectively (Fig. 1C). The 5-year graft survival rate was significantly higher in the IFLT group than in the CLT group after adjustment for donor and recipient risk factors (HR 0.307, 95% CI 0.131–0.719; p <0.01). The unadjusted result remained essentially the same (HR 0.308, 95% CI 0.133–0.716; p <0.01) (Table S2). IFLT recipients had a lower incidence of liver-related graft loss (5.26% *vs*. 30.00%; p <0.01). In the IFLT group, liver-related graft loss was attributed to tumor recurrence (n = 1) and chronic rejection (n = 1). In the CLT group, liver-related graft loss occurred because of primary non-function (PNF) (n = 4), hepatic artery thrombosis (HAT) (n = 7), tumor recurrence (n = 27), and chronic rejection (n = 1). The incidence of non-liver-related graft loss was comparable between the two groups (10.53% *vs*. 13.08%; p = 0.889). Finally, one patient in each group underwent re-transplantation (Table 1). In addition, the 5-year graft survival rate censored for tumor-related graft loss was still significantly superior in the IFLT compared with the CLT group (92.03% *vs*. 77.12%; HR 0.342, 95% CI 0.118–0.996; p = 0.049) (Fig. 1D).

Risk factors of patient survival

Risk factors associated with patient death were further explored. In the univariate analysis, donor age, donor types (extended *vs*. standard criteria), transplant technique (IFLT *vs*. CLT), and recipients' principal diagnosis (HCC *vs*. decompensated cirrhosis) affected patient survival (p <0.05). Then, risk

Table 1. Outcomes and complications.

Outcome parameter	IFLT (n = 38)	CLT (n = 130)	*p* value
Biliary complications, n (%)			0.63
Cholangitis	0	7 (5.38)	0.317
Non-anastomotic strictures	0	3 (2.31)	0.804
PTCD + ERCP + endoscopic stenting	0	1 (0.77)	>0.999
Long-term use of choleretic drugs	0	2 (1.54)	>0.999
Anastomotic strictures	5 (13.16)	20 (15.38)	0.734
Bile leak	1 (2.63)	4 (3.08)	>0.999
Biliary stone	2 (5.26)	6 (4.62)	0.977
Acute rejection, n (%)	7 (18.42)	39 (30.00)	0.159
Infection, n (%)			
Pneumonia	4 (10.53)	23 (17.69)	0.290
Sepsis	3 (7.89)	9 (6.92)	0.878
Other infection	5 (13.16)	11 (8.46)	0.474
Re-transplantation, n (%)	1 (2.63)	1 (0.77)	0.402
Cause of graft loss, n (%)			0.078
Liver-related graft loss	2 (5.26)	39 (30.00)	0.004
Primary non-function	0	4 (3.08)	0.575
Hepatic artery thrombosis	0	7 (5.38)	0.317
Tumor recurrence	1 (2.63)	27 (20.77)	0.008
Rejection	1 (2.63)	1 (0.77)	0.402
Non-liver-related graft loss	4 (10.53)	17 (13.08)	0.889
Cause of patient death, n (%)			0.007
Graft failure	0 (0)	11 (8.46)	0.138
Tumor recurrence	1 (2.63)	28 (21.54)*	0.007
New-onset tumor	1 (2.63)	0	0.226
Sepsis/infection	2 (5.26)	12 (9.23)	0.656
Miscellaneous†	1 (2.63)	5 (3.85)	>0.999

The Chi-square test or Fisher's exact test was used to calculated *p* values, with statistical significance defined as $p < 0.05$.
CLT, conventional liver transplantation; ERCP, endoscopic retrograde cholangiopancreatography; HCC, hepatocellular carcinoma; IFLT, ischemia-free liver transplantation; PTCD, percutaneous transhepatic cholangiography.
*One patient underwent re-transplantation because of hepatic artery thrombosis (the cause of graft loss) and subsequently succumbed to HCC recurrence (the cause of patient death). In total, 25 patients died from HCC recurrence, one from hepatic epithelioid hemangioendothelioma recurrence, and two from cholangiocarcinoma recurrence.
†Miscellaneous included one case of heart failure, one vehicle accident, one head trauma, one suicide in the CLT group, and one case of acute pulmonary embolism in the IFLT group. The cause of death of one patient in the CLT group was unclear, as the patient's family refused to provide further information.

factors with a *p* value <0.1 in the univariate analysis were included in the multivariate analysis. The use of the IFLT technique was an independent protective factor for long-term patient survival (HR 0.246, 95% CI 0.098–0.620; $p < 0.01$). Donor age (HR 1.022, 95% CI 1.001–1.040; $p < 0.05$), HCC before transplantation (HR 2.039, 95% CI 1.159–3.590; $p < 0.05$), and extended criteria donor (HR 2.088, 95% CI 1.215–3.590; $p < 0.01$) were independent risk factors for long-term patient survival (Table 2).

Patient and graft survival in patients with HCC

It was found that HCC before transplantation was a critical independent factor affecting patient survival. Next, we detailed our analysis considering characteristics that define the tumor stage.

In the subgroup analysis of patients with HCC, we observed more patients exceeding the Milan criteria in the CLT group. Thus, we performed a Cox regression analysis to adjust for HCC risk factors between the two groups, although all baseline characteristics did not reach statistical significance ($p > 0.05$) (Table S3). As displayed in Fig. 2, the 5-year overall patient and graft survival rates of the IFLT group were significantly superior to those of the CLT group (82.35% *vs*. 42.03%; HR 0.249, 95% CI 0.074–0.831; $p < 0.05$; and 76.47% *vs*. 42.03%; HR 0.368, 95% CI 0.127–1.060; $p = 0.065$). Moreover, the 5-year recurrence-free survival rate of IFLT recipients was also better than those of CLT recipients (76.47% *vs*. 33.33%; HR 0.324, 95% CI 0.110–0.955; $p < 0.05$). Donor (type) and recipient (maximal tumor diameter, number of tumors, and tumor stages outside of the Milan and University of California San Francisco [UCSF] criteria) risk factors were covariates in the Cox regression analysis. The main reason for the higher survival rate in the IFLT group was the significantly lower incidence of tumor recurrence (5.88% *vs*. 36.23%; $p < 0.05$) (Table S4). Detailed anatomical locations and time intervals of recurrences post transplantation leading to death in patients with HCC are presented in Table S5.

Risk factors associated with patient survival with HCC were explored in univariate and multivariate analyses (Table 3). In the univariate analysis, transplant techniques (IFLT *vs*. CLT), maximal tumor diameter, number of tumors, and tumor stages outside of the Milan or UCSF criteria affected the patient survival ($p < 0.05$). Risk factors with *p* value <0.1 were included in the multivariate analysis. We found that the IFLT technique was an independent factor in improving long-term patient survival (HR 0.249, 95% CI 0.074–0.831; $p < 0.05$), whereas extended criteria donors (HR 2.066, 95% CI 1.079–3.957; $p < 0.05$) and maximal tumor diameter >30 mm (HR 3.109, 95% CI 1.294–7.470; $p < 0.05$) were independent risk factors.

Liver-related complications

Symptomatic NASs occurred exclusively in the CLT group, with no cases in the IFLT group (Table 1).[5] Among three patients with NAS in the CLT group, one received endoscopic stenting and two required long-term use of choleretic drugs. Cholangitis also occurred only in the CLT group and required antibiotic

Table 2. Univariate and multivariate analyses of factors impacting patient survival.

	Univariate analysis		Multivariate analysis*	
	**HR (95% CI)**	***p* value**	**HR (95% CI)**	***p* value**
**Donor characteristics**				
Age (years)	1.024 (1.004–1.045)	0.022	1.022 (1.001–1.040)	0.040
Sex				
Male	Reference	NA		
Female	0.930 (0.519–1.665)	0.806		
BMI (kg/m^2)	1.038 (0.933–1.154)	0.492		
Causes of death				
Head trauma	Reference	NA		
Anoxia	0.399 (0.098–1.634)	0.201		
Cerebrovascular accident	1.379 (0.834–2.278)	0.210		
Miscellaneous	1.975 (0.716–5.448)	0.189		
Donor risk index	2.532 (0.822–7.804)	0.106		
Type				
Standard criteria donor	Reference	NA		
Extended criteria donor†	2.044 (1.204–3.469)	0.008	2.088 (1.215–3.590)	0.008
**Recipient characteristics**				
Age (years)	1.010 (0.985–1.035)	0.443		
Sex				
Male	Reference	NA		
Female	0.521 (0.163–1.663)	0.271		
HBV infection				
Negative	Reference	NA		
Positive	1.250 (0.594–2.629)	0.557		
Waiting time	0.997 (0.991–1.003)	0.334		
Laboratory MELD score‡	1.004 (0.973–1.035)	0.821		
Transplant techniques				
CLT	Reference	NA		
IFLT	0.252 (0.101–0.628)	0.003	0.246 (0.098–0.620)	0.003
Principal diagnosis				
Decompensate cirrhosis	Reference	NA		
HCC	2.487 (1.433–4.319)	0.001	2.039 (1.159–3.590)	0.013
Liver failure	0.517 (0.222–1.201)	0.125		
Miscellaneous	2.062 (0.645–6.597)	0.223		
**Operation characteristics**				
Anhepatic phase (min)	0.999 (0.985–1.013)	0.905		
Recipient operation time (min)				
≤600	Reference	NA		
>600	1.455 (0.527–4.010)	0.469		
Blood loss (ml)				
≤2000	Reference	NA		
>2000	1.255 (0.757–2.080)	0.378		
Intraoperative use of RBCs (ml)				
≤2000	Reference	NA		
>2000	1.398 (0.757–2.580)	0.285		
Intraoperative use of FFP (ml)				
≤2000	Reference	NA		
>2000	0.926 (0.523–1.639)	0.792		

Univariate and multivariate analyses were performed using Cox regression. Statistical significance was set at $p < 0.05$.
CLT, conventional liver transplantation; FFP, fresh frozen plasma; HCC, hepatocellular carcinoma; HR, hazard ratio; IFLT, ischemia-free liver transplantation; INR, international normalized ratio; MELD, model for end-stage liver disease; NA, not applicable; NMP, normothermic machine perfusion; RBC, red blood cell; UNOS, United Network for Organ Sharing.
*Variables with a p value <0.1 in the univariate analysis were included in the multivariate analysis.
†Extended criteria donor was defined as meeting at least one of the following criteria: (1) donor age >60 years; (2) hypernatremia (serum Na^+ >165 mmol/L); (3) >30% macrovesicular steatosis by biopsy; (4) donor serum aspartate aminotransferase or alanine aminotransferase >1000 IU/L or total bilirubin >3 mg/dl before procurement; or (5) cold ischemia time ≥12 h. Detailed specific criteria fulfilled by each extended criteria donor in this study have been provided in the published article.[11]
‡Laboratory MELD score was calculated using the formula 3.8[Ln serum bilirubin (mg/dl)] + 11.2[Ln INR] + 9.6[Ln serum creatinine (mg/dl)] + 6.4, as described by the UNOS for liver transplant prioritization.

therapy. Additional biliary complications including anastomotic strictures, bile leaks, and bile stones were comparable between the groups.

Acute rejection occurred less frequently in the IFLT group, with 7 out of 38 recipients (18.42%), compared with 39 out of 130 recipients (30.00%) in the CLT group. In addition, there were comparable infection rates between the two groups, including pneumonia, sepsis, and other types of infections (Table 1).

Liver function tests

Results of post-transplant liver and renal biochemical blood tests are shown by year in Fig. S2. Some indicators reflecting

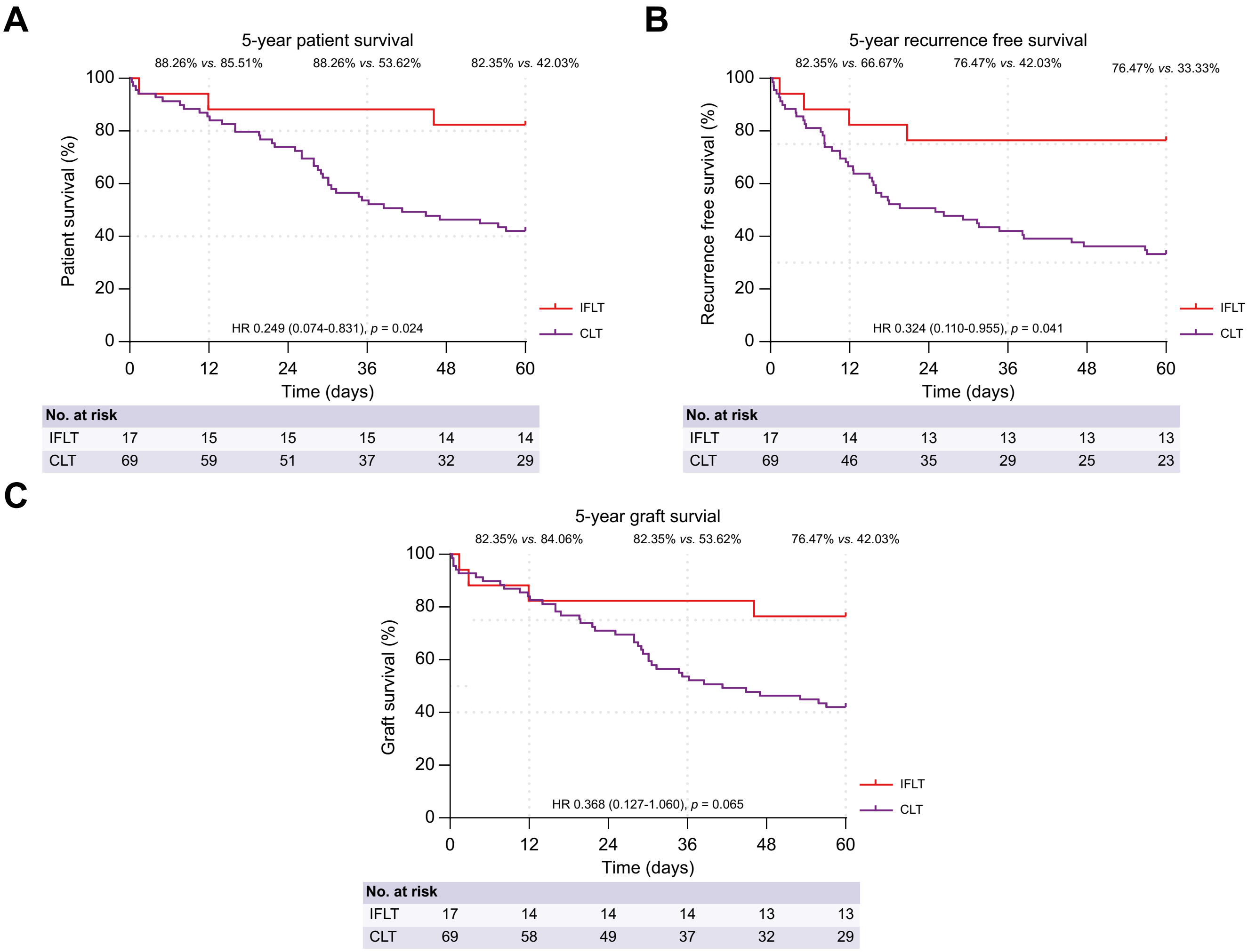

Fig. 2. Five-year survival after liver transplantation in patients with HCC. Time-to-event Kaplan–Meier curves for (A) 5-year patient survival, (B) recurrence-free survival, and (C) graft survival after liver transplantation in patients with HCC. The HR and *p* value in (A)–(C) were calculated using the Cox regression adjusted for donor (type) and recipient (maximal tumor diameter, number of tumors, and tumor stages outside of the Milan and UCSF criteria) risk factors. Statistical significance was set at p <0.05. CLT, conventional liver transplantation; IFLT, ischemia-free liver transplantation; HCC, hepatocellular carcinoma; HR, hazard ratio.

the severity of acute IRI, including ALT, AST, and TBIL, were significantly lower in the IFLT group within 1 week post-operatively. However, these differences gradually disappeared during long-term follow-up. IFLT recipients had a long-term lower trend of ALP and GGT levels, which are related to bile duct injury, but the differences did not reach statistical significance. Other parameters, including ALT, AST, TBIL, GLU, PA, and CREA, were comparable between the groups during long-term follow-up.

Discussion

This is the first 5-year follow-up study on the IFLT technique, demonstrating that its benefits are not limited to reducing early-onset IRI-related complications but also contribute to improving patient and graft survival.

Previous studies have shown that IFLT can reduce the incidence of IRI-related complications, including EAD, post-reperfusion syndrome, asymptomatic NAS, and a 12-month comprehensive complication index.[11–13] These studies mainly used the incidence of EAD as the primary endpoint during a 1-year follow-up. However, the latest consensus on liver machine perfusion argues against EAD as a good surrogate for long-term graft and patient survival, which are the gold standard for assessing clinical benefit.[18] Recently, the results of 5-year follow-ups on NMP and HOPE have been reported and have shown that these techniques can improve 5-year graft survival rates compared with CLT using static cold storage in high-risk donor liver transplants including ECD-DBD, DCD, and discarded livers.[19–23] In the current study, we show for the first time that IFLT improves 5-year patient and graft survival in recipients of DBD liver transplants.

The survival benefit of the IFLT technique is mainly explained by significantly reduced liver-related graft loss (5.26% *vs*. 34.82%) as a consequence of PNF, HAT, and tumor recurrence. Previous studies have confirmed that IRI, as an inevitable component of liver transplantation, could lead to PNF or even patient death in severe cases.[3] By using IFLT, graft IRI can be largely avoided.[10] Moreover, compared with NMP preservation alone, viability tests can be done more quickly and accurately because there is no ischemia before and after *ex situ* NMP preservation during IFLT.[24,25] Therefore, the occurrence

Table 3. Univariate and multivariate analyses of factors contributing to patient survival in recipients with HCC.

	Univariate analysis		Multivariate analysis*	
	HR (95% CI)	*p* value	HR (95% CI)	*p* value
**Donor characteristics**				
Age (years)	1.011 (0.987–1.037)	0.362		
Sex				
Male	Reference	NA		
Female	0.871 (0.439–1.729)	0.693		
BMI (kg/m^2)	0.956 (0.835–1.094)	0.513		
Cause of death				
Head trauma	Reference	NA		
Anoxia	NA	0.997		
Cerebrovascular accident	1.186 (0.651–2.159)	0.577		
Miscellaneous	1.266 (0.306–5.239)	0.745		
Donor risk index	0.799 (0.193–3.307)	0.757		
Type				
Standard criteria donor	Reference	NA		
Extended criteria donor	1.778 (0.957–3.303)	0.068	2.066 (1.079–3.957)	0.029
**Recipient characteristics**				
Age (years)	0.997 (0.967–1.028)	0.852		
Sex				
Male	Reference	NA		
Female	0.742 (0.180–3.070)	0.681		
Laboratory MELD score†	1.006 (0.972–1.041)	0.721		
Transplant techniques				
CLT	Reference	NA		
IFLT	0.232 (0.072–0.751)	0.015	0.249 (0.074–0.831)	0.024
HBV infection				
Negative	Reference	NA		
Positive	1.250 (0.594–2.629)	0.557		
Waiting time	0.997 (0.990–1.005)	0.491		
Bridging or downstaging therapy				
No	Reference	NA		
Hepatectomy	1.158 (0.358–3.744)	0.807		
LRTs	1.157 (0.623–2.148)	0.645		
Hepatectomy and LRTs	1.100 (0.433–2.797)	0.841		
LRTs and TKIs	1.300 (0.313–5.390)	0.718		
AFP (μg/L)				
≤400	Reference	NA		
>400	1.726(0.868–3.432)	0.120		
Maximal tumor diameter (mm)				
≤30	Reference	NA		
>30	3.031 (1.345–6.828)	0.007	3.109 (1.294–7.470)	0.011
Number of tumors				
≤3	Reference	NA		
>3	2.262 (1.239–4.131)	0.008	1.516 (0.702–3.273)	0.290
Partial hepatectomy				
No	Reference	NA		
Yes	1.135 (0.526–2.447)	0.747		
Milan criteria				
In	Reference	NA		
Out	2.443 (1.168–5.113)	0.018	0.430 (0.089–2.079)	0.294
UCSF criteria				
In	Reference	NA		
Out	2.963 (1.486–5.906)	0.002	3.076 (0.673–14.064)	0.147
Child–Pugh class				
A	Reference	NA		
B	0.688 (0.367–1.288)	0.242		
C	1.101 (0.511–2.375)	0.806		
BCLC stage				
0	Reference	NA		
A	0.908 (0.447–1.845)	0.790		
B	1.176 (0.613–2.257)	0.626		
C	1.308 (0.670–2.550)	0.431		
D	1.101 (0.511–2.375)	0.806		
ECOG score				
0	Reference	NA		
1	0.973 (0.383–2.474)	0.955		
2	NA	0.996		

(*continued on next page*)

Table 3. (*continued*)

	Univariate analysis		Multivariate analysis*	
	HR (95% CI)	*p* value	HR (95% CI)	*p* value
**Immunosuppressive regimen**				
MMF				
No	Reference	NA		
Yes	0.677 (0.367–1.248)	0.211		
mTOR				
No	Reference	NA		
Yes	0.733 (0.395–1.361)	0.325		
**Operation characteristics**				
Anhepatic phase (min)	1.001 (0.984–1.018)	0.948		
Recipient operation time (min)				
≤600	Reference	NA		
>600	0.978 (0.236–4.047)	0.976		
Blood loss (ml)				
≤2,000	Reference	NA		
>2,000	0.977 (0.516–1.849)	0.943		
Intraoperative use of RBCs (ml)				
≤2,000	Reference	NA		
>2,000	1.315 (0.406–4.254)	0.648		
Intraoperative use of FFP (ml)				
≤2,000	Reference	NA		
>2,000	0.966 (0.448–2.082)	0.929		

Univariate and multivariate analyses were performed using Cox regression. Statistical significance was set at p <0.05.
AFP, alpha-fetoprotein; BCLC, Barcelona Clinic Liver Cancer; CLT, conventional liver transplantation; ECOG, Eastern Cooperative Oncology Group Performance Status; FFP, fresh frozen plasma; HCC, hepatocellular carcinoma; HR, hazard ratio; IFLT, ischemia-free liver transplantation; INR, international normalized ratio; LRT, locoregional therapy; MELD, model for end-stage liver disease; MMF, mycophenolate mofetil; mTOR, mammalian target of rapamycin; NA, not applicable; RBC, red blood cell; TKI, tyrosine kinase inhibitor; UCSF, University of California San Francisco; UNOS, United Network for Organ Sharing.
*Variables with a p value <0.1 in the univariate analysis were included in the multivariate analysis.
†Laboratory MELD score was calculated using the formula 3.8[Ln serum bilirubin (mg/dl)] + 11.2[Ln INR] + 9.6[Ln serum creatinine (mg/dl)] + 6.4, as described by the UNOS for liver transplant prioritization.

of PNF can be avoided using IFLT. Interestingly, the incidence of HAT was reduced in the IFLT *vs*. CLT recipients, which might be explained by the use of a donor celiac artery for anastomosis and activation of fibrinolysis during *ex situ* NMP.[26–28] Therefore, IFLT can improve graft survival censored for tumor-related death and patient survival censored for tumor-related graft loss by avoiding PNF and reducing HAT.

Importantly, IRI can affect and alter the immune microenvironment of the graft, making it more susceptible to tumor recurrence.[29] Clinical studies have shown that severe IRI is associated with a higher rate of tumor recurrence in transplant recipients with HCC.[15,30,31] Previous studies have shown that IRI triggers cancer recurrence through CXCL10/CXCR3 signaling to mobilize regulatory T cells.[32] Our previous experimental study has shown that IFLT can largely abrogate IRI.[10] Moreover, the CXCR3 and cytokine–cytokine receptor interaction pathways are downregulated in the IFLT grafts. In the current study, the results of subgroup Cox regression analysis show that both patient overall survival and recurrence-free survival are improved in patients with HCC receiving IFLT after adjustment for HCC risk factors, including HCC stages. Moreover, our results show that IFLT can substantially reduce cancer recurrence rate compared with CLT, which contributes to its long-term survival benefit. Data from a national cohort study showed that less than 40% of patients with HCC met the Milan criteria at the time of transplant in China.[33] For patients with HCC exceeding the Milan criteria, the 5-year recurrence rate exceeds 40%, compared with less than 20% for those within the Milan criteria.[34] The high proportion of cases beyond the Milan criteria in our study likely contributed to the observed high recurrence and low survival rates in the CLT group. Notably, the CLT group had a slightly higher proportion of patients exceeding the Milan criteria, which may have exaggerated the survival difference between the groups. To address this, we performed Cox regression and multivariate analyses, adjusting for key HCC risk factors. The adjusted results continued to support the advantages of IFLT in reducing recurrence rates and improving long-term survival, particularly in high-risk patients with HCC, likely by mitigating the effects of graft IRI on cancer recurrence.

Allograft rejection is also a major cause of late graft loss.[35] Studies have shown that IRI can trigger alloimmune responses by reactive oxygen species production, which induces damage-associated molecular patterns, leading to dendritic cell activation and the subsequent initiation of adaptive alloimmunity.[36] Several studies have demonstrated that novel preservation techniques ameliorate the consequences of IRI with a reduced risk of acute rejection.[20,37,38] Our experimental study has shown that redox hemostasis was maintained in IFLT grafts but not in CLT grafts.[10] In the current study, we also documented a reduction in rejection rates among IFLT recipients.

Undoubtedly, there are some limitations to this study. First, because of the limited availability of disposable sets and perfusionists for machine perfusion, we initially designed a non-randomized trial to evaluate the safety and effectiveness of IFLT. Although donor and recipient characteristics were balanced between groups, selection bias cannot be entirely ruled out. To this end, a multivariate Cox proportional hazards regression was further performed to adjust for potential baseline differences. Second, the outcomes reported in this study were not the primary endpoints designed in the original study

protocol, which limits multiple testing for numerous parameters. Hence, the analysis of these outcomes should be considered exploratory. Third, patients in this study were from a single center, and the sample size was relatively small, especially in the subgroup analysis, which may limit the generalizability of the findings. Nevertheless, our data are unique and based on an entirely novel approach that improves outcomes significantly while serving as a platform for detailed mechanistic studies. Lastly, additional procurement time and the seamless collaboration of the multiorgan procurement teams are required to ensure the procurement of other donor organs via the DBD procedure when IFLT is conducted.

In conclusion, the results of the current study demonstrate the potential long-term benefits of IFLT in improving 5-year patient and graft survival, thus serving as a basis for future multicenter randomized trials.

Affiliations

[1]*Organ Transplant Center, The First Affiliated Hospital, Sun Yat-sen University, Guangzhou, China;* [2]*Guangdong Provincial Key Laboratory of Organ Medicine, Guangzhou, China;* [3]*Guangdong Provincial International Cooperation Base of Science and Technology (Organ Transplantation), Guangzhou, China;* [4]*State Key Laboratory of Ophthalmology, Zhongshan Ophthalmic Center, Sun Yat-sen University, Guangzhou, China;* [5]*Transplantation Center, Digestive Disease and Surgery Institute and Department of Immunology, Lerner Research Institute, Cleveland Clinic, Cleveland, OH, USA;* [6]*Division of Transplant Surgery, Brigham and Women's Hospital, Harvard Medical School, Boston 02115, MA, USA;* [7]*NHC Key Laboratory of Assisted Circulation, Sun Yat-sen University, Guangzhou, China*

Abbreviations

ALP, alkaline phosphatase; ALT, alanine aminotransferase; AST, aspartate aminotransferase; CLT, conventional liver transplantation; CREA, creatinine; DBD, donation after brain death; DCD, donation after cardiac death; EAD, early allograft dysfunction; GGT, gamma-glutamyltransferase; GLU, glucose; HAT, hepatic artery thrombosis; HCC, hepatocellular carcinoma; HOPE, hypothermic oxygenated perfusion; HR, hazard ratio; IFLT, ischemia-free liver transplantation; IRI, ischemia–reperfusion injury; NAS, non-anastomotic stricture; NMP, normothermic machine perfusion; PA, prealbumin; PNF, primary non-function; RCT, randomized controlled trial; TBIL, total bilirubin; UCSF, University of California San Francisco.

Financial support

This study was supported through the following grants: the National Natural Science Foundation of China (81970564, 82070670, 82170663, 82370664, and 82300744), the Guangdong Provincial Key Laboratory Construction Projection on Organ Donation and Transplant Immunology (2023B1212060020), the Guangdong Provincial International Cooperation Base of Science and Technology (Organ Transplantation) (2020A0505020003), and the Science and Technology Program of Guangdong (2024B1515040011). This study was also supported by the China Organ Transplantation Development Foundation. The funders/sponsors had no role in the design and conduct of the study; collection, management, analysis, and interpretation of the data; preparation, review, or approval of the manuscript; and decision to submit the manuscript for publication.

Conflicts of interest

The authors of this study declare that they do not have any conflict of interest.

Please refer to the accompanying ICMJE disclosure forms for further details.

Authors' contributions

Had full access to all of the data in the study and take responsibility for the integrity of the data and the accuracy of the data analysis: XH, ZG. Had access to the trial results and reviewed and approved the final version of the manuscript for publication: all authors. Served as the principal investigator for the study: XH, ZG. Contributed to study design: XH, ZG, ZJ, CH. Drafted the protocol: XH, ZG, ZJ, CH. Enrolled the patients and conducted the operations: TW, ZC, YT, QZ, YC, MC, AH, WJ, YM, DW, XZ. Collected the data: JZ, NZ, SL. Analyzed the data: ZJ, JZ, NZ, SL, YD, YL. Interpreted the data: XH, ZG, ZJ, JZ, NZ, SL, YD, YL. Drafted the manuscript: XH, ZG, ZJ. Participated in paper writing and editing: ZJ, AS, SGT.

Data availability statement

Data can be made available to researchers upon reasonable request.

Acknowledgements

We express our gratitude to the patients who consented to participate in this trial, as well as to the doctors, coordinators, operating room staff, and organ perfusionists for their invaluable contributions to this study.

Supplementary data

Supplementary data to this article can be found online at https://doi.org/10.1016/j.jhepr.2025.101393.

References

Author names in bold designate shared co-first authorship

[1] Morris PJ. Transplantation—a medical miracle of the 20th century. N Engl J Med 2004;351:2678–2680.

[2] Feng S, Goodrich NP, Bragg-Gresham JL, et al. Characteristics associated with liver graft failure: the concept of a donor risk index. Am J Transpl 2006;6:783–790.

[3] **de Rougemont O**, **Dutkowski P**, Clavien P-A. Biological modulation of liver ischemia-reperfusion injury. Curr Opin Organ Transpl 2010;15:183–189.

[4] **Ceresa CDL**, **Nasralla D**, Pollok JM, et al. Machine perfusion of the liver: applications in transplantation and beyond. Nat Rev Gastroenterol Hepatol 2022;19:199–209.

[5] van Rijn R, Schurink IJ, de Vries Y, et al. Hypothermic machine perfusion in liver transplantation—a randomized trial. N Engl J Med 2021;384:1391–1401.

[6] Schlegel A, Mueller M, Muller X, et al. A multicenter randomized-controlled trial of hypothermic oxygenated perfusion (HOPE) for human liver grafts before transplantation. J Hepatol 2023;78:783–793.

[7] Markmann JF, Abouljoud MS, Ghobrial RM, et al. Impact of portable normothermic blood-based machine perfusion on outcomes of liver transplant: the OCS Liver PROTECT randomized clinical trial. JAMA Surg 2022;157:189–198.

[8] Nasralla D, Coussios CC, Mergental H, et al. A randomized trial of normothermic preservation in liver transplantation. Nature 2018;557:50–56.

[9] **He X**, **Guo Z**, **Zhao Q**, et al. The first case of ischemia-free organ transplantation in humans: a proof of concept. Am J Transpl 2018;18:737–744.

[10] **Guo Z**, **Xu J**, **Huang S**, et al. Abrogation of graft ischemia-reperfusion injury in ischemia-free liver transplantation. Clin Transl Med 2022;12:e546.

[11] **Guo Z**, **Zhao Q**, **Huang S**, et al. Ischaemia-free liver transplantation in humans: a first-in-human trial. Lancet Reg Health West Pac 2021;16:100260.

[12] **Guo Z**, **Zhao Q**, **Jia Z**, et al. A randomized-controlled trial of ischemia-free liver transplantation for end-stage liver disease. J Hepatol 2023;79:394–402.

[13] **Chen M**, **Chen Z**, **Lin X**, et al. Application of ischaemia-free liver transplantation improves prognosis of patients with steatotic donor livers—a retrospective study. Transpl Int 2021;34:1261–1270.

[14] **Wang S**, **Lin X**, **Tang Y**, et al. Ischemia-free liver transplantation improves the prognosis of recipients using functionally marginal liver grafts. Clin Mol Hepatol 2024;30:421–435.

[15] **Tang Y**, **Wang T**, **Ju W**, et al. Ischemic-free liver transplantation reduces the recurrence of hepatocellular carcinoma after liver transplantation. Front Oncol 2021;11:773535.

[16] Huang JF, Wang HB, Zheng SS, et al. Advances in China's organ transplantation achieved with the guidance of law. Chin Med J (Engl) 2015;128:143–146.

[17] **de Vries Y**, **von Meijenfeldt FA**, Porte RJ. Post-transplant cholangiopathy: classification, pathogenesis, and preventive strategies. Biochim Biophys Acta Mol Basis Dis 2018;1864(4 Pt B):1507–1515.

[18] Martins PN, Rizzari MD, Ghinolfi D, et al. Design, analysis, and pitfalls of clinical trials using ex situ liver machine perfusion: the International Liver

Transplantation Society Consensus Guidelines. Transplantation 2021;105:796–815.
[19] Czigany Z, Uluk D, Pavicevic S, et al. Improved outcomes after hypothermic oxygenated machine perfusion in liver transplantation-Long-term follow-up of a multicenter randomized controlled trial. Hepatol Commun 2024;8:e0376.
[20] Mergental H, Laing RW, Kirkham AJ, et al. Discarded livers tested by normothermic machine perfusion in the VITTAL trial: secondary end points and 5-year outcomes. Liver Transpl 2024;30:30–45.
[21] **Schlegel A**, **Muller X**, Kalisvaart M, et al. Outcomes of DCD liver transplantation using organs treated by hypothermic oxygenated perfusion before implantation. J Hepatol 2019;70:50–57.
[22] Yamamoto T, Atthota S, Agarwal D, et al. Impact of portable normothermic machine perfusion for liver transplantation from adult deceased donors. Ann Surg 2023;278:e922–e929.
[23] van Leeuwen OB, Bodewes SB, Porte RJ, et al. Excellent long-term outcomes after sequential hypothermic and normothermic machine perfusion challenges the importance of functional donor warm ischemia time in DCD liver transplantation. J Hepatol 2023;79:e244–e245.
[24] **Guo Z**, **Zhan L**, **Gao N**, et al. Metabolomics differences of the donor livers between in situ and ex situ conditions during ischemia-free liver transplantation. Transplantation 2023;107:e139–e151.
[25] **Zhang Z**, **Tang Y**, **Zhao Q**, et al. Association of perfusion characteristics and posttransplant liver function in ischemia-free liver transplantation. Liver Transpl 2020;26:1441–1454.
[26] Algarni AA, Mourad MM, Bramhall SR. Anticoagulation and antiplatelets as prophylaxis for hepatic artery thrombosis after liver transplantation. World J Hepatol 2015;7:1238–1243.
[27] Dar WA, Sullivan E, Bynon JS, et al. Ischaemia reperfusion injury in liver transplantation: cellular and molecular mechanisms. Liver Int 2019;39:788–801.
[28] Karangwa SA, Burlage LC, Adelmeijer J, et al. Activation of fibrinolysis, but not coagulation, during end-ischemic ex situ normothermic machine perfusion of human donor livers. Transplantation 2017;101:e42–e48.
[29] **Uehara M**, **Solhjou Z**, Banouni N, et al. Ischemia augments alloimmune injury through IL-6-driven $CD4^+$ alloreactivity. Sci Rep 2018;8:2461.
[30] Ling Q, Liu J, Zhuo J, et al. Development of models to predict early post-transplant recurrence of hepatocellular carcinoma that also integrate the quality and characteristics of the liver graft: a national registry study in China. Surgery 2018;164:155–164.
[31] **Mueller M**, **Kalisvaart M**, **O'Rourke J**, et al. Hypothermic oxygenated liver perfusion (HOPE) prevents tumor recurrence in liver transplantation from donation after circulatory death. Ann Surg 2020;272:759–765.
[32] **Li CX**, **Ling CC**, Shao Y, et al. CXCL10/CXCR3 signaling mobilized-regulatory T cells promote liver tumor recurrence after transplantation. J Hepatol 2016;65:944–952.
[33] Hu L, Zhao Z, Mu F, et al. Utilization of elderly donors in liver transplantation for patients with hepatocellular carcinoma: a national retrospective cohort study of China. Int J Surg 2022;105:106839.
[34] Tabrizian P, Holzner ML, Mehta N, et al. Ten-year outcomes of liver transplant and downstaging for hepatocellular carcinoma. JAMA Surg 2022;157:779–788.
[35] Abbasoglu O, Levy MF, Brkic BB, et al. Ten years of liver transplantation: an evolving understanding of late graft loss. Transplantation 1997;64:1801–1807.
[36] Land WG. Emerging role of innate immunity in organ transplantation: part I: evolution of innate immunity and oxidative allograft injury. Transpl Rev (Orlando) 2012;26:60–72.
[37] Czigany Z, Pratschke J, Fronek J, et al. Hypothermic oxygenated machine perfusion reduces early allograft injury and improves post-transplant outcomes in extended criteria donation liver transplantation from donation after brain death: results from a multicenter randomized controlled trial (HOPE ECD-DBD). Ann Surg 2021;274:705–712.
[38] Maspero M, Ali K, Cazzaniga B, et al. Acute rejection after liver transplantation with machine perfusion versus static cold storage: a systematic review and meta-analysis. Hepatology 2023;78:835–846.

Keywords: Liver transplantation; Ischemia–reperfusion injury; Ischemia-free organ transplantation; Long-term outcomes.
Received 13 January 2025; received in revised form 5 March 2025; accepted 7 March 2025; Available online 12 March 2025

Journal of Hepatology, Volume *7*

Supplemental information

Ischemia-free liver transplantation improves long-term outcomes in a 5-year follow-up study

Zehua Jia, Jiaxing Zhu, Jiayi Zhang, Jian Zhang, Changjun Huang, Niancun Zhang, Songming Li, Yuqi Dong, Yao Liu, Ping Zeng, Tielong Wang, Zhitao Chen, Yunhua Tang, Qiang Zhao, Maogen Chen, Yinghua Chen, Anbin Hu, Weiqiang Ju, Yi Ma, Dongping Wang, Xiaofeng Zhu, Andrea Schlegel, Tullius G. Stefan, Xiaoshun He, and Zhiyong Guo

Ischemia-free liver transplantation improves long-term outcomes in a 5-year follow-up study

Zehua Jia, Jiaxing Zhu, Jiayi Zhang, Jian Zhang, Changjun Huang, Niancun Zhang, Songming Li, Yuqi Dong, Yao Liu, Ping Zeng, Tielong Wang, Zhitao Chen, Yunhua Tang, Qiang Zhao, Maogen Chen, Yinghua Chen, Anbin Hu, Weiqiang Ju, Yi Ma, Dongping Wang, Xiaofeng Zhu, Andrea Schlegel, Tullius G. Stefan, Xiaoshun He, Zhiyong Guo

Table of contents

Supplementary methods……………………………………………………………….2

Supplementary figures………………………………………………………….…...3

Supplementary tables…………………………………………………………….....5

Supplementary methods

Post-transplant immunosuppression protocol

Standardized post-transplant immunosuppressive treatment was provided for both groups. The immunosuppression (IS) therapy was divided into induction and maintenance phases as follows:

- Induction phase: Basiliximab (20mg) was administered intravenously during the operation and at post-operative day (POD) 4.
- Maintenance phase: The maintenance therapy began at POD 4. The mainstay of maintenance therapy is the combined use of tacrolimus and mycophenolic acid or sirolimus. The initial dose of tacrolimus was 0.04 mg/kg/d, and the target trough level was 8-10 ng/ml within the first three months, and 6-8 ng/ml thereafter.

Post-transplant tumor recurrence surveillance protocol for patients with liver cancer

Standardized post-transplant tumor recurrence surveillance was implemented. This included regular monitoring of alpha-fetoprotein (AFP) levels and imaging studies:

- AFP Monitoring: Conducted monthly during the first year and every 2-3 months from the second to fifth year post-transplantation.
- Imaging (CT/MRI/ultrasound): Performed every 6 months during the first year and annually from the second to fifth year post-transplantation.

Additionally, AFP levels and imaging studies were promptly conducted for any patients presenting with abnormal clinical signs or test results suggestive of tumor recurrence. This consistent surveillance ensured the reliable detection of tumor recurrence in both groups.

Supplementary figures

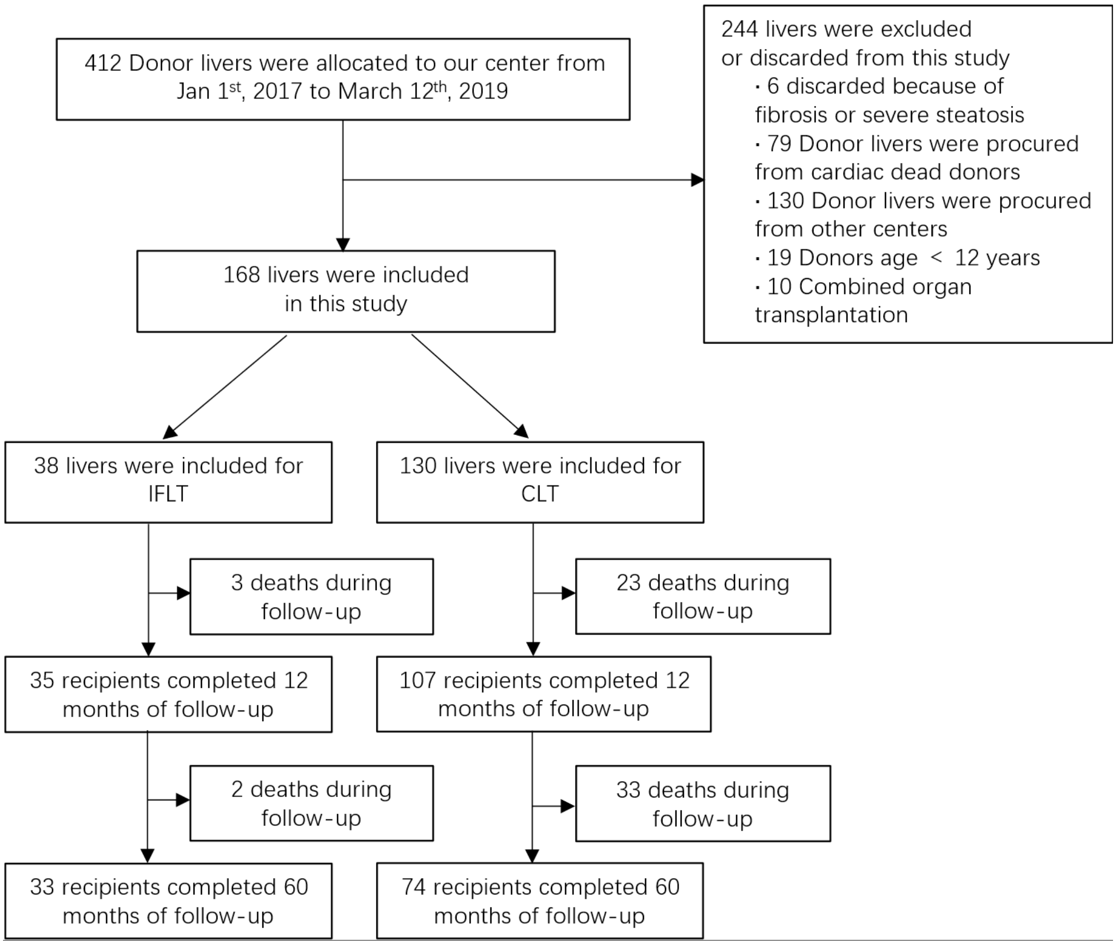

Fig. S1. Flow diagram of patient screening, selection and follow-up.

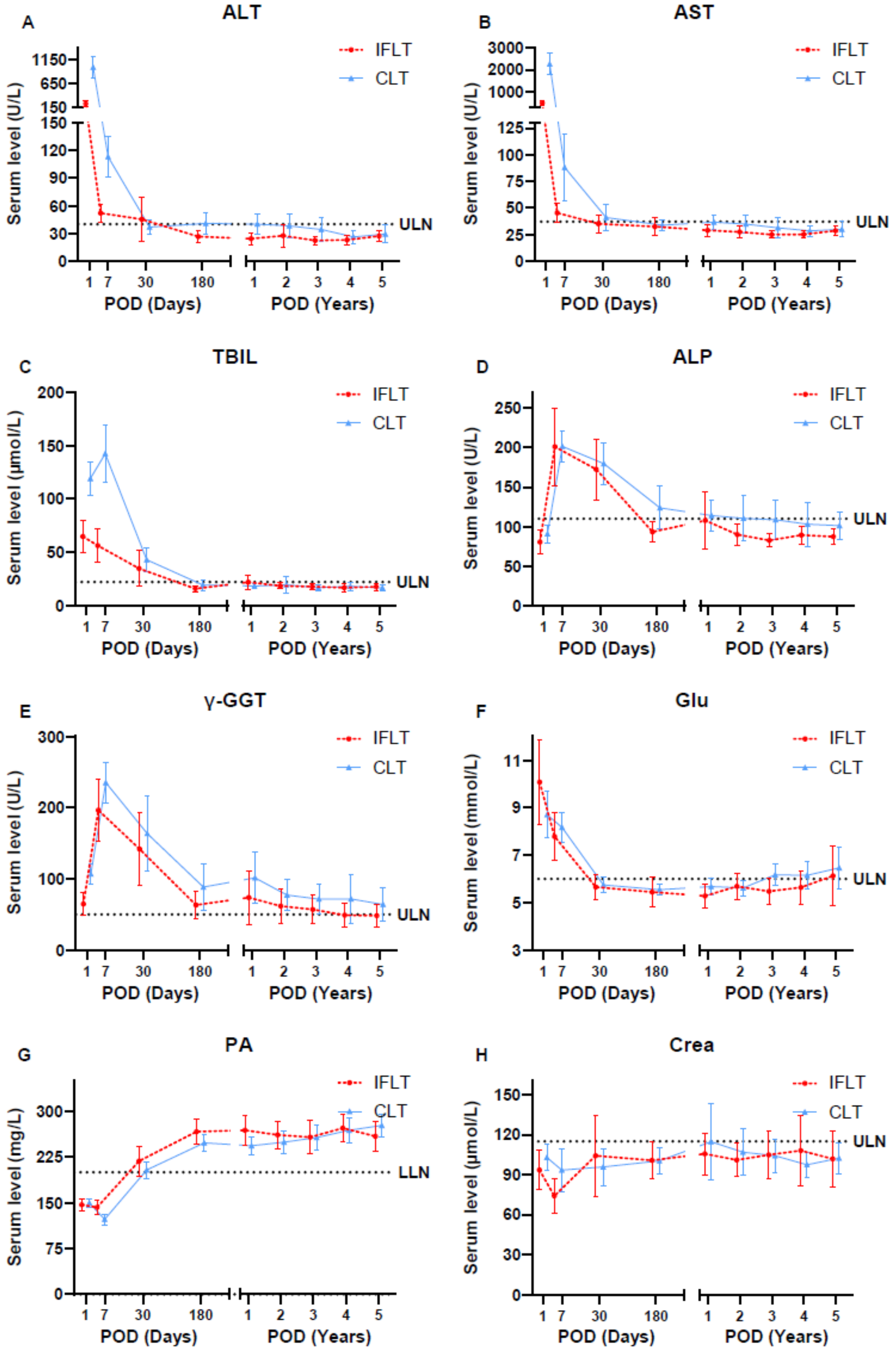

Fig. S2. Liver function tests after liver transplantation.

Continuous variables are presented as mean and 95%CI (Confidence Interval). IFLT, ischemia-free liver transplantation; CLT, conventional liver transplantation; ULN, upper limit of normal; LLN, lower limit of normal; PA, prealbumin; TBIL, total bilirubin; Glu, glucose; Crea, creatinine; γGGT, gamma-glutamyl transferase; LDH, lactate dehydrogenase; ALP, alkaline phosphatase; ALT, alanine aminotransferase; AST, aspartate aminotransferase.

Supplementary tables

Table S1. Baseline demographic and clinical characteristics of donors and recipients.

	IFLT (n=38)	CLT (n=130)	*p* value
**Donor characteristics**			
Age, mean (SD), years	36 (14)	37 (12)	0.597
Male, No. (%)	31 (81.58)	94 (72.31)	0.347
BMI, mean (SD)	22.27 (2.16)	22.47 (2.45)	0.648
Cause of death, No. (%)			0.161
Head trauma	21 (55.26)	55 (42.31)	
Anoxia	3 (7.89)	9 (6.92)	
Cerebrovascular accident	11 (28.95)	62 (47.69)	
Miscellaneous†	3 (7.89)	4 (3.08)	
Donor risk index, mean (SD)	1.34 (0.24)	1.36 (0.21)	0.656
Extended criteria donor, No. (%)‡	12 (31.58)	29 (22.31)	0.339
Macrosteatosis, No. (%)			0.643
No/Mild (<30%)	36 (94.74)	125 (96.15)	
Moderate (30%-60%)	1 (2.63)	4 (3.08)	
Severe (>60%)	1 (2.63)	1 (0.77)	
**Recipient characteristics**			
Age, mean (SD), years	51 (11)	50 (10)	0.741
Male, No. (%)	34 (89.47)	120 (92.31)	0.824
Laboratory MELD score, mean (SD) §	23.94 (6.69)	24.58 (8.62)	0.670
HBV positive, No. (%)	32 (84.21)	110 (84.62)	1.000
Waiting time, mean (SD), days	30 (49)	31 (60)	0.885
Principal diagnosis, No. (%)			0.182
HCC	17 (44.74)	69 (53.08)	
Decompensate cirrhosis	16 (42.11)	32 (24.62)	
Liver failure	4 (10.53)	25 (19.23)	
Miscellaneous¶	1 (2.63)	4 (3.08)	
**Operation characteristics**			
Liver retrieval time, median (IQR), min	204 (190-220)	45 (40-59)	<0.001
NMP duration time, median (IQR), min	240 (160-360)	NA	NA
Cold ischemia time, median (IQR), min	NA	369 (329-450)	NA
Anhepatic time, mean (SD), min	52 (15)	53 (20)	0.706
Recipient operation time > 600 min, No. (%)	0	9 (6.92)	0.208
Blood loss > 2000 ml, No. (%)	16 (42.11)	53 (40.77)	1.000
Intraoperative use of RBCs > 2000 ml, No. (%)	4 (10.53)	27 (20.77)	0.232
Intraoperative use of FFP > 2000 ml, No. (%)	10 (26.32)	37 (28.46)	0.957

* Continuous variables were compared using t-test or the Mann-Whitney U test, while categorical variables were analyzed using the chi-square test and Fisher's exact test. Statistical significance was set at $p < 0.05$. IFLT, ischemia-free liver transplantation; CLT, conventional liver transplantation; BMI, body mass index; MELD, model for end-stage liver disease; HBV, hepatitis B virus; HCC, hepatocellular carcinoma; NMP, normothermic machine perfusion; RBC, red blood cell; FFP, fresh frozen plasma.

† Miscellaneous includes bacterial encephalitis, viral encephalitis, organophosphorus poisoning.

‡ Extended criteria donor (ECD) was defined as meeting at least one of the following criteria: (1) donor age >60 years; (2) hypernatremia (serum Na^+ >165 mmol/L); (3) >30% macrovesicular steatosis by biopsy; (4) donor serum aspartate aminotransferase (AST) or alanine amino- transferase (ALT) >1,000 IU/L or total bilirubin (Tbil) >3 mg/dL before procurement; (5) or cold ischemia time (CIT) ≥12 hours.

§ Laboratory MELD score was calculated using the formula: 3.8 [Ln serum bilirubin (mg/dL)] + 11.2[Ln INR] + 9.6[Ln serum creatinine (mg/dL)] + 6.4, as described by the United Network for Organ Sharing (UNOS) for liver transplant prioritization.

¶ Miscellaneous includes Budd-Chiari syndrome, cholangiocarcinoma, hepatic mucinous cystic neoplasm, hepatic epithelioid hemangioendothelioma.

Table S2. Cox regression analysis of 5-year patient and graft survival.

Outcomes	Hazard ratio (95% CI)	*p* value
5-year patient survival		
Unadjusted	0.252 (0.101-0.628)	0.003
Adjusted*	0.246 (0.098-0.620)	0.003
5-year patient survival (censored for tumor related death)		
Unadjusted	0.324 (0.098-1.070)	0.064
Adjusted*	0.244 (0.072-0.825)	0.023
5-year graft survival		
Unadjusted	0.308 (0.133-0.716)	0.006
Adjusted*	0.307 (0.131-0.719)	0.007
5-year patient graft (censored for tumor related graft loss)		
Unadjusted	0.422 (0.148-1.200)	0.110
Adjusted*	0.342 (0.118-0.996)	0.049

* The hazard ratio (HR) and *p* value were calculated using the Cox regression adjusted for donor (age and type) and recipient (HCC prior to transplantation) risk factors. Statistical significance was set at $p < 0.05$.

Table S3. Donor, recipient and operation characteristics in patients with HCC prior to transplantation.*

	IFLT (n=17)	CLT (n=69)	*p* value
**Donor characteristics**			
Age, mean (SD), years	34 (12)	38 (12)	0.197
Male, No. (%)	13 (76.47)	49 (71.01)	0.883
BMI, mean (SD)	22.22 (2.05)	22.63 (2.29)	0.501
Cause of death, No. (%)			0.182
Head trauma	10 (58.82)	30 (43.48)	
Anoxia	1 (5.88)	4 (5.80)	
Cerebrovascular accident	4 (23.53)	33 (47.83)	
Miscellaneous†	2 (11.76)	2 (2.90)	
Donor risk index, mean (SD)	1.29 (0.17)	1.36 (0.21)	0.239
Extended criteria donor, No. (%)	7 (41.18)	19 (27.54)	0.423
Macrosteatosis, No. (%)			0.643
No/Mild (<30%)	15 (88.24)	66 (95.65)	
Moderate (30%-60%)	1 (5.88)	3 (4.35)	
Severe (>60%)	1 (5.88)	0 (0)	
**Recipient characteristics**			
Age, mean (SD), years	51 (11)	51 (10)	0.894
Male, No. (%)	16 (94.12)	65 (94.20)	1.000
HBV positive, No. (%)	15 (88.2)	66 (95.7)	0.554
Waiting time, mean (SD), days	39 (67)	27 (38)	0.347
Bridging or downstaging therapy			0.159
No	11 (64.71)	28 (40.58)	
Hepatectomy	1 (5.88)	5 (7.25)	
LRTs	2 (11.76)	27 (39.13)	
Hepatectomy and LRTs	3 (17.65)	6 (8.70)	
LRTs and TKIs	0 (0)	3 (4.35)	
Laboratory MELD score, mean (SD)‡	20.47 (5.02)	24.14 (8.98)	0.109
AFP >400 μg/L, No. (%)	3 (17.65)	14 (20.29)	1.000
Maximal tumor diameter > 30 mm, No. (%)	9 (52.94)	49 (71.01)	0.256
Number of tumors > 3, No. (%)	4 (23.53)	28 (40.58)	0.306
In Milan criteria, No. (%)	8 (47.06)	21 (30.43)	0.311
In UCSF criteria, No. (%)	10 (58.82)	27 (39.13)	0.232
Child-Pugh class, No. (%)			0.242
A	4 (23.53)	30 (43.48)	
B	8 (47.06)	28 (40.58)	
C	5 (29.41)	11 (15.94)	
BCLC stage, No. (%)			0.753
0	1 (5.88)	4 (5.80)	
A	3 (17.65)	17 (24.64)	
B	5 (29.41)	20 (28.99)	
C	3 (17.65)	17 (24.64)	
D	5 (29.41)	11 (15.94)	
ECOG score, No. (%)			0.097
0	13 (76.47)	61 (88.41)	
1	3 (17.65)	8 (11.59)	
2	1 (5.88)	0	
Immunosuppressive regimen, No. (%)			
CNIs	17 (100.00)	69 (100.00)	1.000
MMF	10 (58.82)	46 (66.67)	0.746
mTOR	5 (29.41)	29 (42.03)	0.499

**Operation characteristics**			
Anhepatic time, mean (SD), min	48 (13)	51 (19)	0.547
Recipient operation time > 600 min, No. (%)	0	4 (5.80)	0.709
Blood loss > 2000 ml, No. (%)	6 (35.29)	22 (31.88)	1.000
Intraoperative use of RBCs > 2000 ml, No. (%)	1 (5.88)	4 (5.80)	1.000
Intraoperative use of FFP > 2000 ml, No. (%)	3 (17.65)	13 (18.84)	1.000

* Continuous variables were compared using t-test or the Mann-Whitney U test, while categorical variables were analyzed using the chi-square test and Fisher's exact test. Statistical significance was set at $p < 0.05$. HCC, hepatocellular carcinoma; IFLT, ischemia-free liver transplantation; CLT, conventional liver transplantation; BMI, body mass index; MELD, model for end-stage liver disease; HBV, hepatitis B virus; TKI, tyrosine kinase inhibitors; LRT, locoregional therapy; AFP, alpha-fetoprotein; UCSF, the University of California, San Francisco criteria; BCLC, the Barcelona Clinic Liver Cancer staging classification; ECOG, Eastern Cooperative Oncology Group Performance Status; CNI, calcineurin inhibitor; MMF, mycophenolate mofetil; mTOR, mammalian target of rapamycin; RBC, red blood cell; FFP, fresh frozen plasma.

† Miscellaneous includes bacterial encephalitis, viral encephalitis, organophosphorus poisoning.

‡ Laboratory MELD score was calculated using the formula: 3.8 [Ln serum bilirubin (mg/dL)] + 11.2[Ln INR] + 9.6[Ln serum creatinine (mg/dL)] + 6.4, as described by the United Network for Organ Sharing (UNOS) for liver transplant prioritization.

Table S4. Causes of death in patients with HCC prior to transplantation.*

Causes of patient death	IFLT (n=17)	CLT (n=69)	*p* value
Tumor recurrence	1(5.88)	25(36.23)	0.02
Hepatic artery thrombosis	0	3(4.35)	1.00
Acute pulmonary embolism	1(5.88)	0	0.20
Primary non-function	0	1(1.45)	1.00
Sepsis/infection	1(5.88)	6(8.70)	1.00
Rejection	0	1(1.45)	1.00
Miscellaneous†	0	4(5.80)	0.60

* The chi-square test or Fisher's exact test was used to calculated *p* values, with statistical significance defined as $p < 0.05$. Data are presented as n (%). IFLT, ischemia-free liver transplantation; CLT, conventional liver transplantation.

† Miscellaneous includes one case of vehicle accident, one hip fracture and head trauma, one suicide in the conventional liver transplantation group only. The cause of death of one patient in the conventional liver transplantation group is unclear as the patient's family refused to provide further information.

Table S5. Detailed anatomical locations and time interval of recurrences post-transplantation leading to death in patients with HCC.*

Number ID	Anatomical locations of recurrences	Time interval of recurrence after transplantation (days)
IFLT-24	liver and lung	621
CLT-8	bile duct	1370
CLT-11	liver	343
CLT-21	liver and lung	376
CLT-23	liver, lymph nodes, retroperitoneal region, pancreas and bone	451
CLT-24	liver and lung	949
CLT-27	lung and bone	54
CLT-28	liver and lung	377
CLT-32	liver	481
CLT-37	lung	788
CLT-43	lung	245
CLT-46	lung	1153
CLT-50	liver	533
CLT-51	Greater omentum and peritoneum	161
CLT-79	lung	589
CLT-80	liver	280
CLT-83	lung	114
CLT-97	liver	14
CLT-105	lung	246
CLT-108	liver and lung	463
CLT-111	liver	44
CLT-112	lung and adrenal gland	876
CLT-113	lung	237
CLT-116	liver	155
CLT-119	liver	67
CLT-124	lung	1147

* IFLT, ischemia-free liver transplantation; CLT, conventional liver transplantation.
